# Supplementary material for: Molecular characterization of Richter syndrome identifies de novo diffuse large B-cell lymphomas with poor prognosis
Source: Nat Commun. 2023 Jan 19;14:309. doi: 10.1038/s41467-022-34642-6 (PMC9852595; doi:10.1038/s41467-022-34642-6)
Supplement: Supplementary file 1 — Supplementary Information [file 41467_2022_34642_MOESM1_ESM.pdf]

# Molecular characterization of Richter syndrome identifies *de novo* diffuse large B-cell lymphomas with poor prognosis

*Running title:* **DNA methylation and gene expression in Richter syndrome**


**Authors:**

Julien Broséus[§,* 1,2,3], Sébastien Hergalant[§ 2], Julia Vogt [4], Eugen Tausch [1], Markus Kreuz [5], Anja Mottok [4], Christof Schneider [1], Caroline Dartigeas [6], Damien Roos-Weil [7], Anne Quinquenel [8], Charline Moulin [9,10], German Ott [11], Odile Blanchet [12], Cécile Tomowiak [13,14], Grégory Lazarian [15], Pierre Rouyer [2], Emil Chteinberg [4], Stephan H. Bernhart [16], Olivier Tournilhac [17], Guillaume Gauchotte [2,18], Sandra Lomazzi [19], Elise Chapiro [20,21], Florence Nguyen-Khac [20,21], Céline Chery [2,22], Frédéric Davi [21,23], Mathilde Hunault [24], Rémi Houlgatte [2], Andreas Rosenwald,[25] Alain Delmer [8], David Meyre [2], Marie-Christine Béné [26,27], Catherine Thieblemont [28], Peter Lichter [29], Ole Ammerpohl [4], Jean-Louis Guéant [2,22], ICGC MMML-Seq Consortium, Romain Guièze [17], José Ignacio Martin-Subero [30,31], Florence Cymbalista [15], Pierre Feugier[# 2,9], Reiner Siebert[# 4] and Stephan Stilgenbauer[#,* 1].

**Affiliations:**

(1) Division of CLL. Department of Internal Medicine III, Ulm University, Ulm, Germany.
(2) Inserm UMRS1256 Nutrition-Génétique et Exposition aux Risques Environnementaux (N-GERE), Université de Lorraine, Nancy, France.
(3) Université de Lorraine, CHRU-Nancy, service d'hématologie biologique, pôle laboratoires, F54000, Nancy, France.
(4) Institute of Human Genetics, Ulm University & Ulm University Medical Center, Ulm, Germany
(5) Fraunhofer Institute for Cell Therapy and Immunology IZI, Leipzig, Germany.
(6) Department of Haematology, University Hospital of Tours, Tours, France.
(7) Department of Hematology, Hôpital de la Pitié-Salpêtrière, AP-HP, Paris, France.
(8) Université de Reims Champagne-Ardenne, IRMAIC, Centre Hospitalier Universitaire de Reims, Hématologie Clinique, Reims, France.
(9) Department of Hematology, University Hospital of Nancy, Vandoeuvre-lès-Nancy, France
(10) Inserm, CHRU, University of Lorraine, CIC Clinical epidemiology, Nancy, France
(11) Department of Clinical Pathology, Robert-Bosch-Krankenhaus, and Dr. Margarete Fischer-Bosch Institute for Clinical Pharmacology, Stuttgart, Germany.
(12) CHU Angers, Biological Resource Center of Angers (CRB-CHU Angers), BB-0033-00038, Laboratoire d'Hématologie, Angers, France.
(13) Department of Hematology, CHU Poitiers, Poitiers, France.
(14) CIC1402 Inserm Poitiers, France.
(15) Hematology Laboratory, Avicenne Hospital, Assistance Publique-Hôpitaux de Paris, Paris, France.
(16) Bioinformatics Group, Department of Computer Science and Interdisciplinary Center for Bioinformatics, Leipzig University, Germany
(17) Hematology department, Clermont-Ferrand University Hospital, Clermont-Ferrand, France.
(18) Department of Biopathology CHRU-ICL, BBB, CHRU Nancy, Vandoeuvre-lès-Nancy, France
(19) Biological Resource Center of Nancy, BB-0033-00035, CHRU de Nancy, Nancy, France.
(20) Sorbonne Université, Cytogénétique Hématologique, Hôpital Pitié-Salpêtrière, AP-HP, Paris, France;

(21) Centre de Recherche des Cordeliers, INSERM, Université Sorbonne Paris Cite, Université Paris Descartes, Université Paris Diderot, F-75006 Paris.
(22) CHRU of Nancy, service de biochimie-biologie moléculaire-nutrition, pôle laboratoires, F54000, Nancy, France.
(23) Hematology Department, Hôpital Pitié-Salpêtrière, AP-HP, Sorbonne University, Paris, France.
(24) Department of Hematology, University Hospital of Angers, Angers, France.
(25) Institute of Pathology, University Hospital of Würzburg, Germany.
(26) Hematology Biology, University Hospital of Nantes, Hôtel-Dieu
(27) Inserm 1232 Centre de Recherche en Cancérologie et Immunologie Nantes Angers (CRCINA), Nantes, France.
(28) Department of Hematology, Hôpital Saint-Louis, Paris, France.
(29) Division of Molecular Genetics, German Cancer Consortium (DKTK) and National Center for Tumor Diseases (NCT) Heidelberg, German Cancer Research Center (DKFZ), Heidelberg, Germany.
(30) Biomedical Epigenomics Group, Institut d'investigacions Biomèdiques August Pi I Sunyer (IDIBAPS), University of Barcelona, Barcelona, Spain.
(31) Institució Catalana de Recerca i Estudis Avançats (ICREA), Barcelona, Spain.

[§] These authors contributed equally
[#] These authors jointly supervised this work
* Corresponding authors

**Corresponding authors:**

Julien Broséus: julien.broseus@univ-lorraine.fr

Stephan Stilgenbauer: Stephan.Stilgenbauer@uniklinik-ulm.de

# Table of contents

**Supplemental figures……5**
**Figure S1……5**
**Figure S2……6**
**Figure S3……7**
**Figure S4……8**
**Figure S5……9**
**Figure S6……10**
**Figure S7……11**
**Figure S8……12**
**Figure S9……13**
**Figure S10……14**
**Figure S11……15**
**Figure S12……16**
**Figure S13……17**
**Figure S14……18**
**Figure S15……19**
**Figure S16……20**
**Figure S17……21**
**Figure S18……22**
**Figure S19……23**
**Figure S20……24**
**Figure S21……25**
**Figure S22……26**
**Figure S23……27**
**Figure S24……28**
**Figure S25……29**
**Figure S26……30**
**Figure S27……31**
**Figure S28……32**
**Figure S29……33**
**Figure S30……34**
**Figure S31……35**
**Figure S32……36**
**Figure S33……37**

**Figure S34……………………………………………………………………………………………38**
**Figure S35……………………………………………………………………………………………..39**
**Figure S36……………………………………………………………………………………………..40**
**Supplemental methods…………………………………………………………………………………..41**
**ICGC MMML-seq consortium members………………………………………………………….......46**
**MMML-seq members………………………………………………………………………………......48**
**References…………………………………………………………………………….......................49**

# Supplemental figures

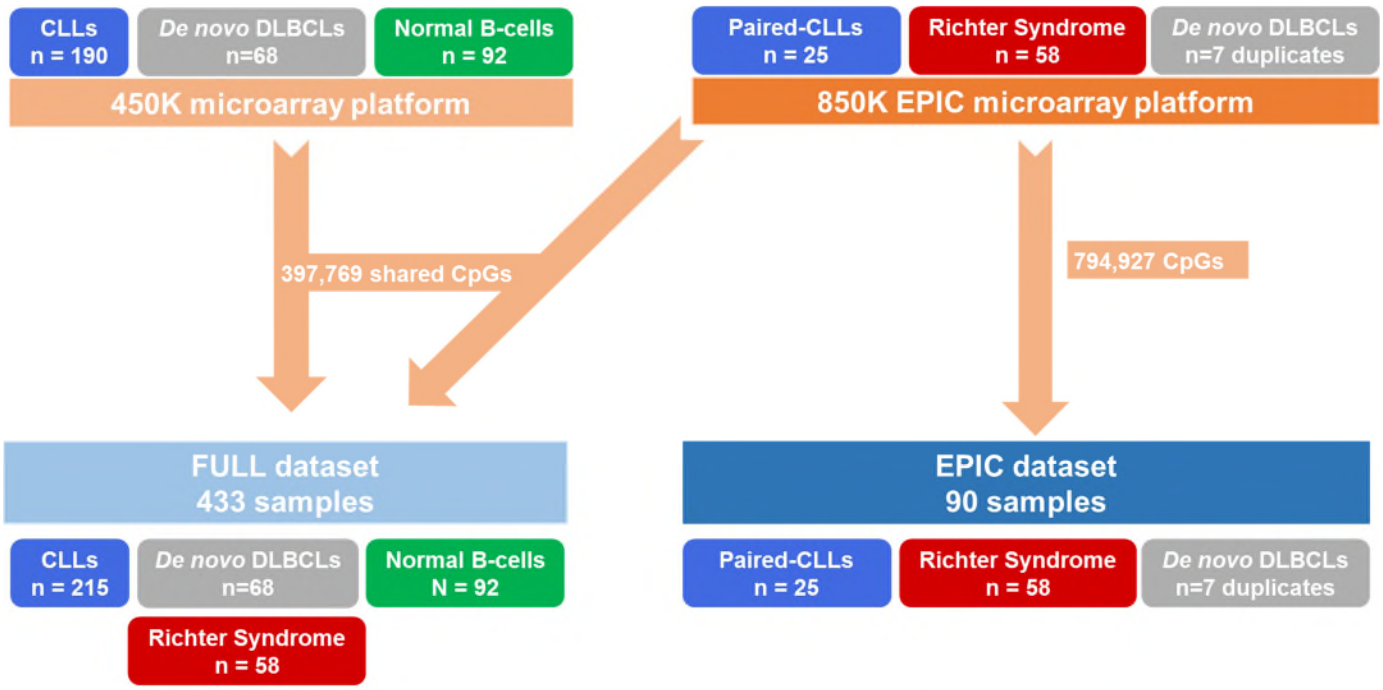


**Supplementary Fig. 1. DNA methylation data analysis workflow.** CLL: chronic lymphocytic leukemia; DLBCL: diffuse large B-cell lymphoma.

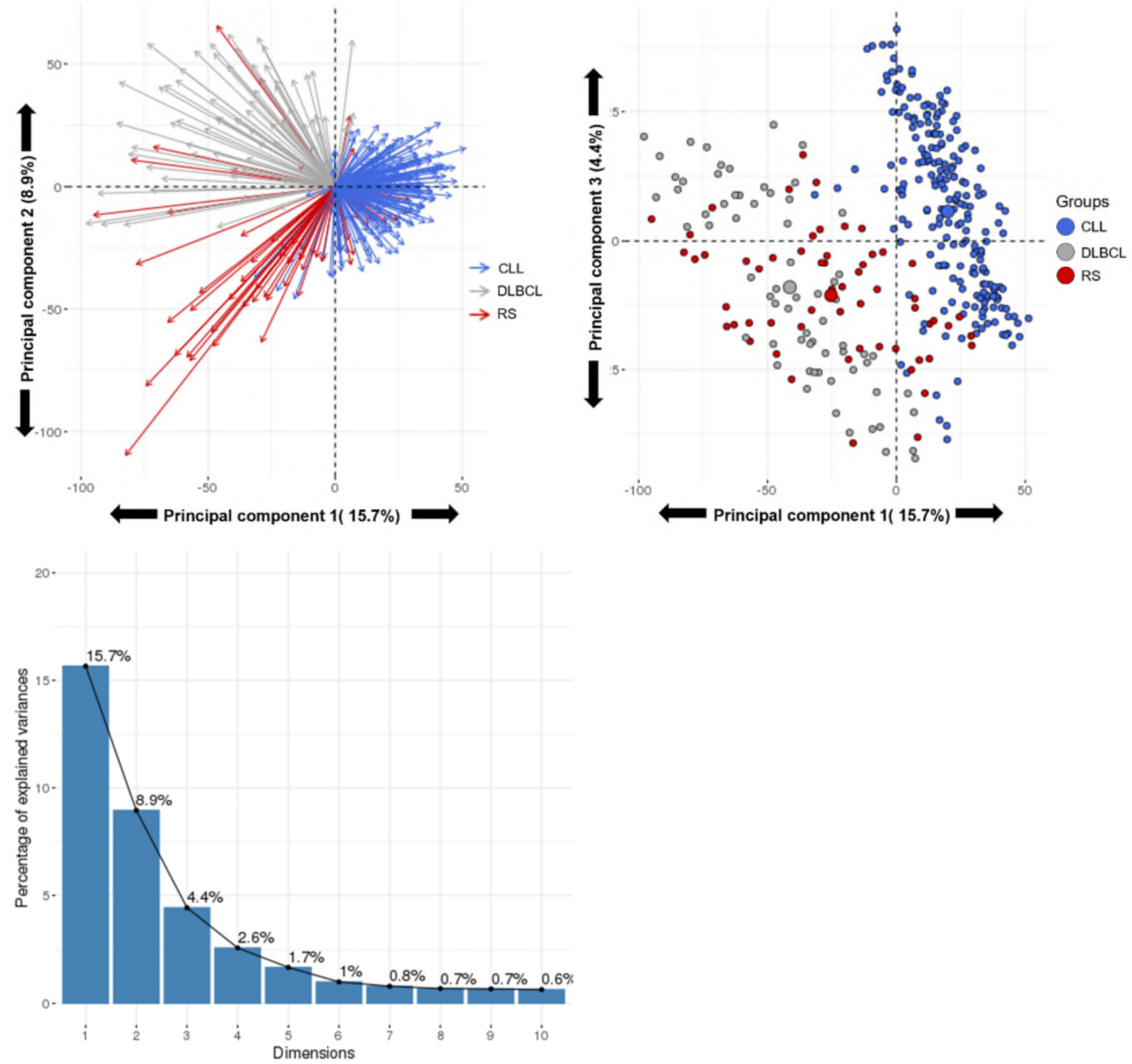


**Supplementary Fig. 2. RS is hypomethylated as compared with *de novo* DLBCL or CLL.** Unsupervised PCA of 58 RS, 215 CLLs and 68 DLBCLs. On PC1: RS is hypomethylated against CLL. On PC2: RS is hypomethylated against DLBCL. On PC3: CLL progression. Other dimensions each explain a small amount of variance (as seen on the scree-plot). For each group, geometrical centers are represented by bigger circles of the same color. CLL: chronic lymphocytic leukemia; DLBCL: *de novo* diffuse large B-cell lymphoma; PCA: principal component analysis; RS: Richter syndrome.

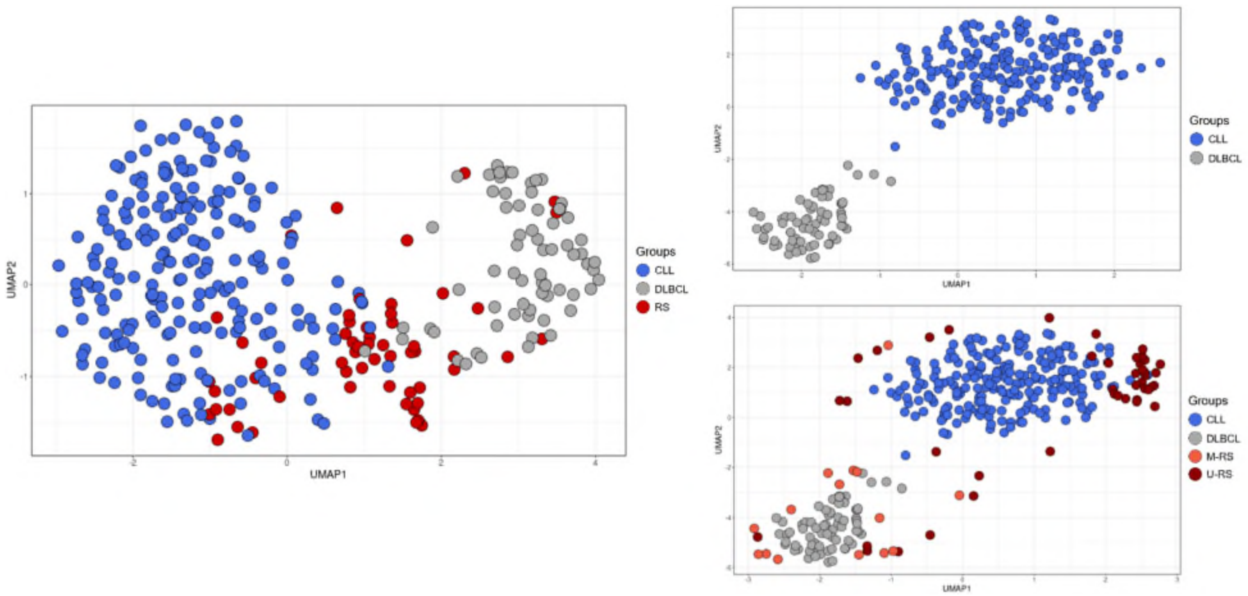


**Supplementary Fig. 3. UMAP of all CpGs from FULL dataset.**
Left part: UMAP applied to the 58 RS, 215 CLL and 68 DLBCL cases, on 397,769 QC-selected CpGs shared by both 450K and EPIC microarray platforms. Right part: UMAP of CLL and *de novo* DLBCLs (top), with predicted scattering of RS cases (bottom), demonstrating that sample distribution is not driven by *IGHV* mutational status. CLL: chronic lymphocytic leukemia; DLBCL: *de novo* diffuse large B-cell lymphoma; M-RS: IGHV-mutated Richter syndrome; RS: Richter syndrome; U-RS: IGHV-unmutated Richter syndrome.

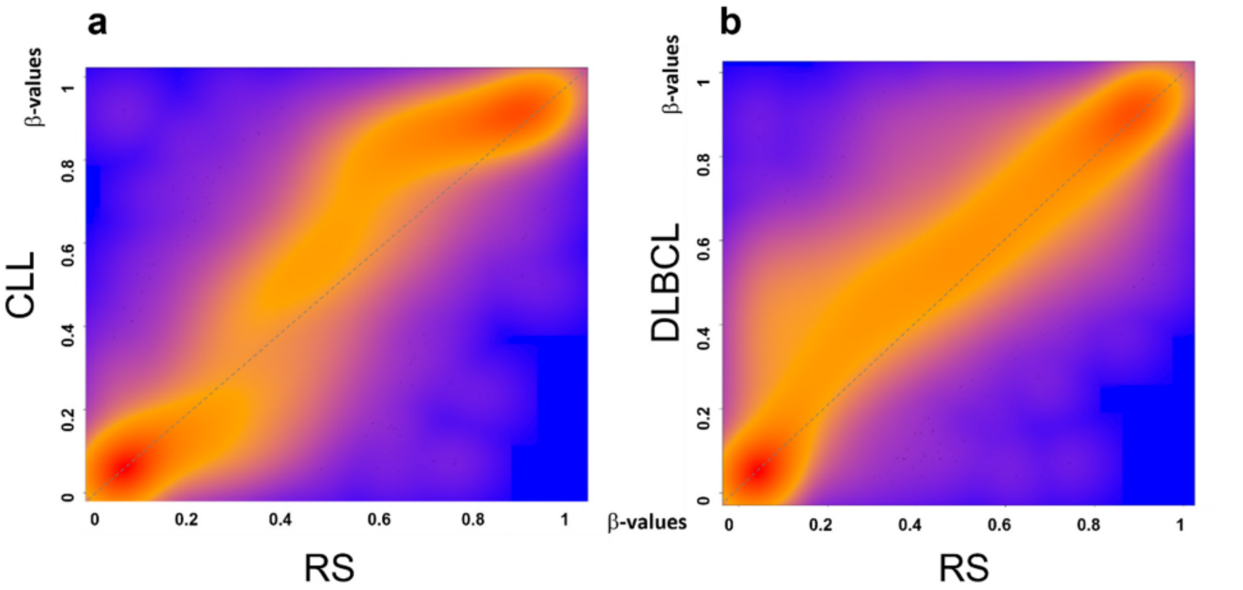


**Supplementary Fig. 4. Comparative smoothed methylation scatterplots between RS and CLL or DLBCL.** Each dot represents one of the 397,769 CpGs in the FULL dataset. For each CpG, coordinates on the x-axis correspond to the mean beta-value for the RS group and coordinates on the y-axis correspond to the mean beta-value for **a** the CLL group or **b** the DLBCL group. Scale from blue (no density) to yellow (medium density) and red (high density), purple and orange are medium-low and medium-high densities, respectively. CLL: chronic lymphocytic leukemia; DLBCL: *de novo* diffuse large B-cell lymphoma; RS: Richter syndrome.

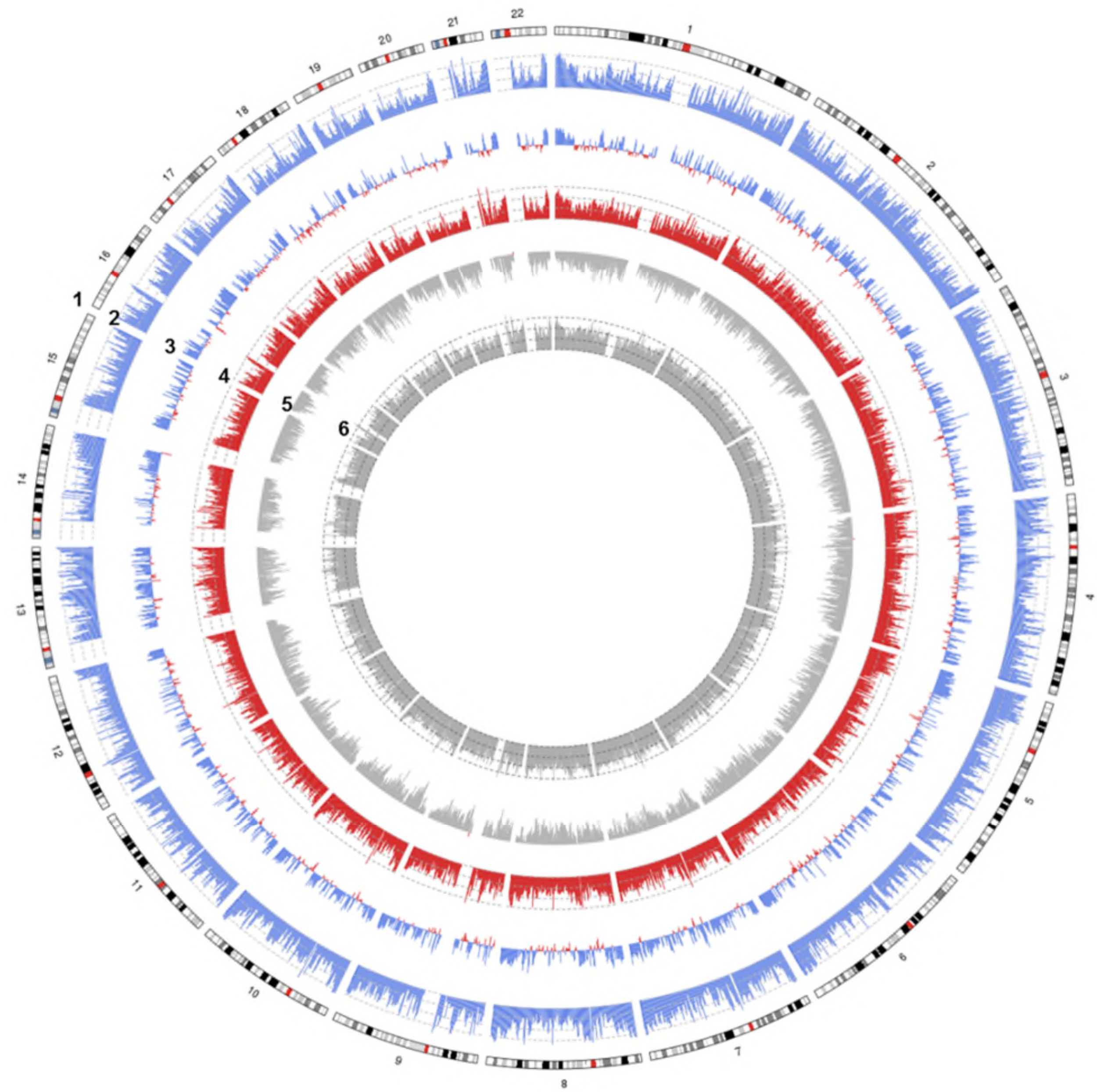


**Supplementary Fig. 5. Circular plot of median CpG methylation levels and differences in CLL (blue), RS (red) and DLBCL (grey) over sliding windows of 500 kb.** RS is hypomethylated as compared with CLL and DLBCLs, but CLL relative hypermethylation is due to localized and highly methylated areas while DLBCL relative hypermethylation is evenly distributed throughout the genome. From outer to inner track: 1) Chromosome number and ideograms with cytobands; 2) CLL methylation levels; 3) CLL minus RS methylation differences. Range from -10% to +30%. Areas hypermethylated in CLL are represented with blue peaks pointing towards the CLL track while areas hypermethylated in RS are represented with red peaks pointing towards the RS track; 4) RS methylation levels; 5) RS minus DLBCL methylation differences (range from -30% to +10%). Areas hypermethylated in RS are represented with red peaks pointing towards the RS track while areas hypermethylated in DLBCLs are represented with grey peaks pointing towards the DLBCL track; 6) DLBCL methylation levels. On tracks 2, 4 and 6, dashed lines represent 0%, 25%, 50%, and 75% average methylation. CLL: chronic lymphocytic leukemia; DLBCL: *de novo* diffuse large B-cell lymphoma; RS: Richter syndrome.

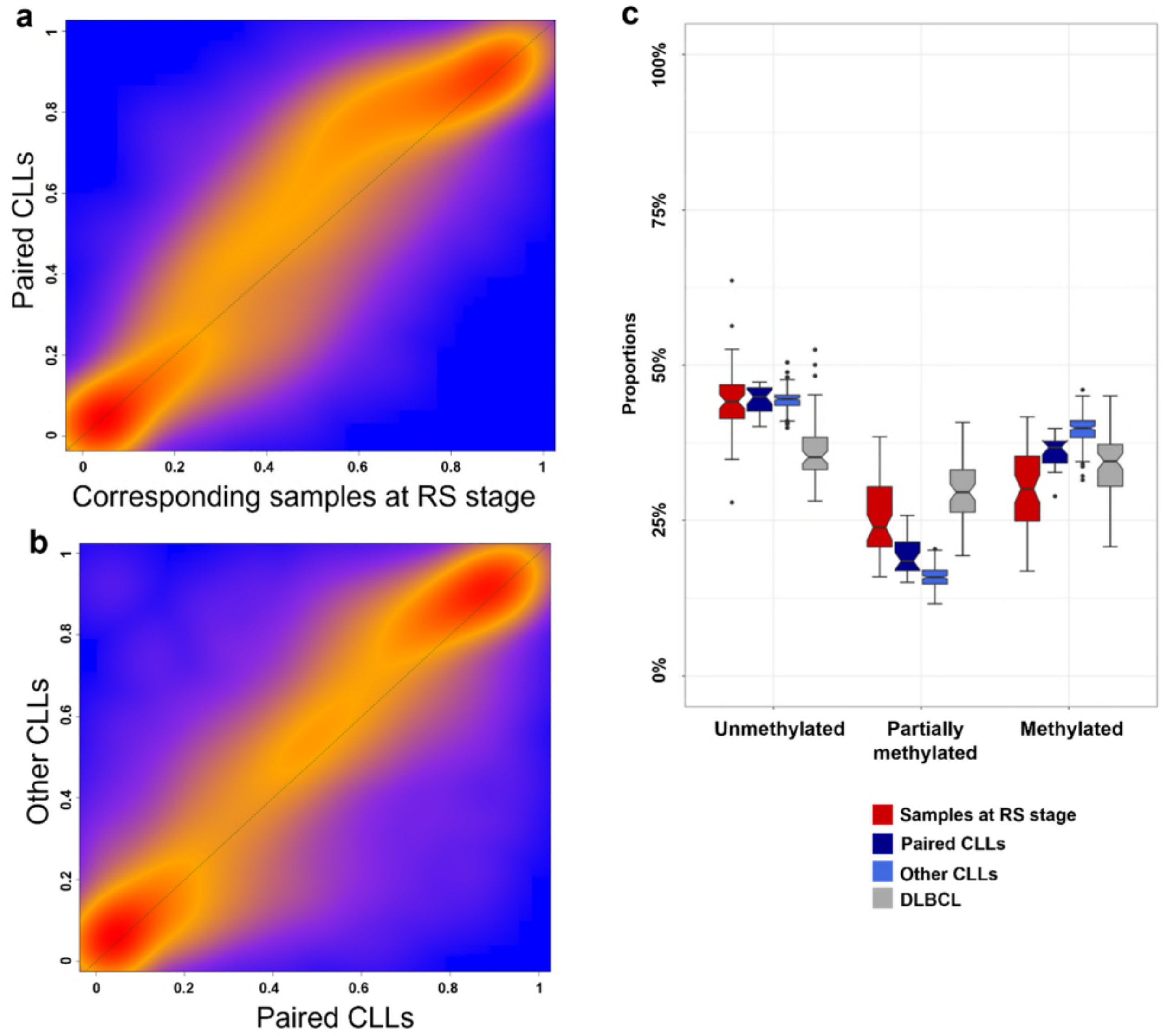


**Supplementary Fig. 6. Comparative smoothed methylation scatterplots between: a** paired CLLs and corresponding samples at RS stage or **b** paired CLLs and other CLLs. Scale from blue (no density) to yellow (medium density) and red (high density). **c** Distribution of unmethylated (beta-value < 0.3), partially methylated (0.3 < beta-value < 0.7) and methylated (beta-value >0.7) CpGs across RS, DLBCL, paired-CLLs and other CLLs, with sample variability. Box plot: the center line, box limits, whiskers and points represent the mean, 25th and 75th percentile, 1.5x interquartile range and individual samples beyond the 1.5x interquartile, respectively. RS: n=58 biologically independent samples; paired-CLLs: n=25 biologically independent samples; other CLLs: n=190 biologically independent samples; DLBCL: n= 68 biologically independent samples. Source data are provided as a Source Data file. CLL: chronic lymphocytic leukemia; DLBCL: *de novo* diffuse large B-cell lymphoma; RS: Richter syndrome.

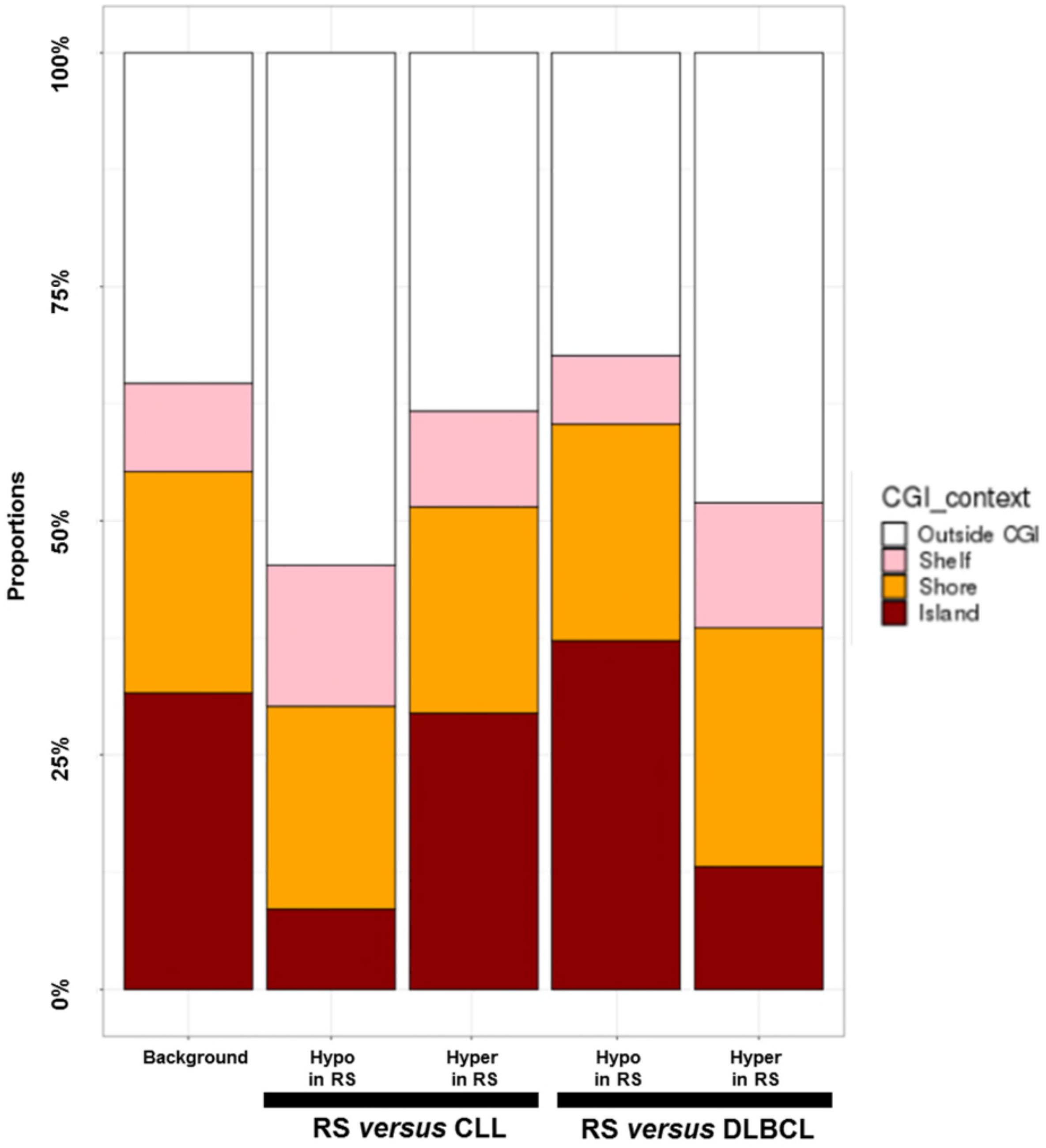


**Supplementary Fig. 7. Location of RS vs CLL and DLBCL differential CpGs regarding regulatory region context.** Distribution of significant and differential CpGs (FDR < 0.01; beta-value differential > 10%) outside CGI and inside CGI (Shelf, Shore, Island). A methylation hallmark of RS is the gain and loss of differential CpGs outside and inside CpG islands, respectively. Distribution bias against background for CGI: CpGs hypomethylated in RS versus CLL were enriched outside CGI and impoverished in Island (p=2.98e-4). CpGs hypermethylated in RS versus DLBCLs were enriched outside CGI and impoverished in Island (p=1.54e-2). CGI: CpG island; CLL: chronic lymphocytic leukemia; DLBCL: *de novo* diffuse large B-cell lymphoma; FDR: false discovery rate; Hyper: hypermethylated; Hypo: hypomethylated; RS: Richter syndrome.

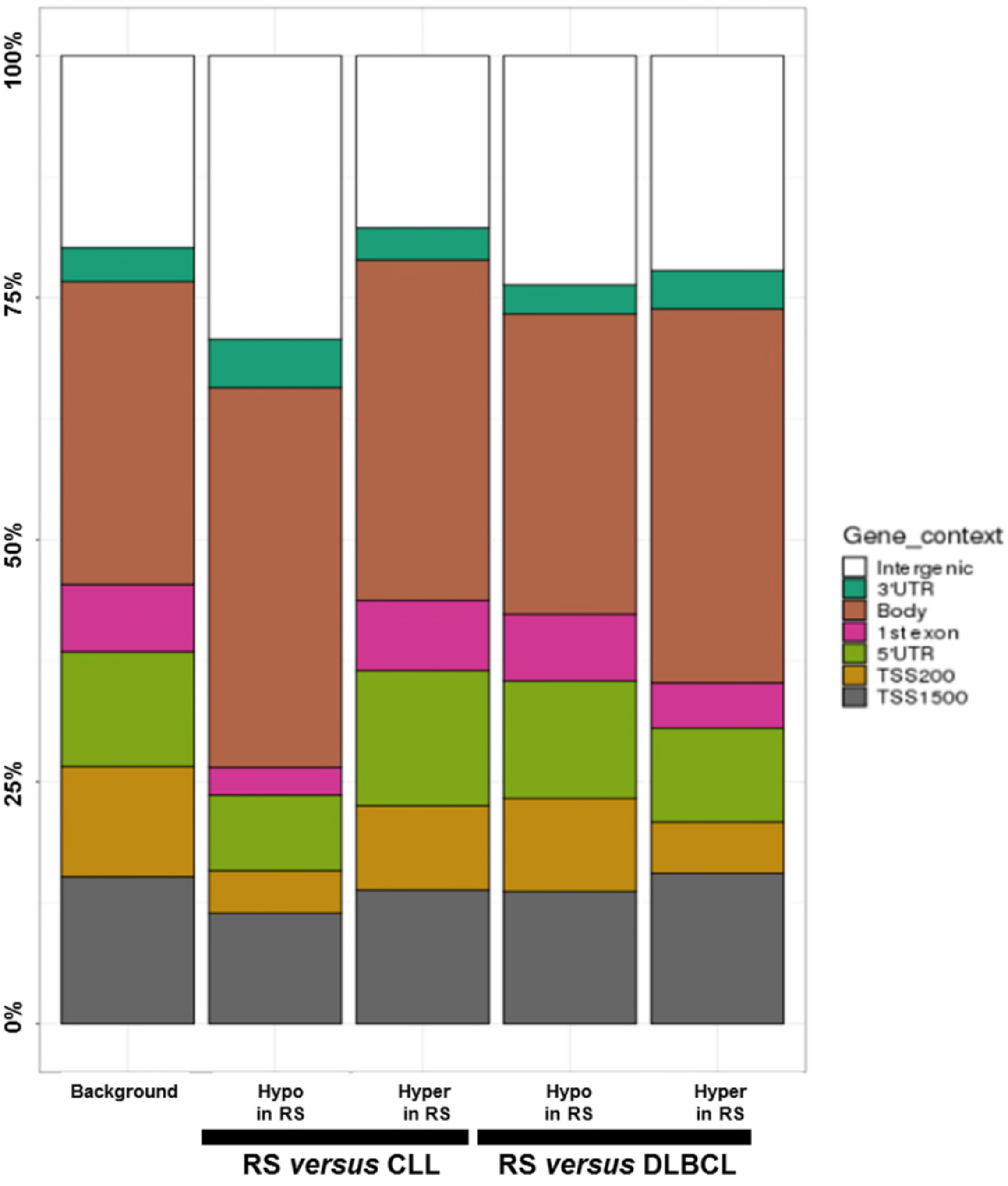


**Supplementary Fig. 8. Location of RS vs CLL and DLBCL differential CpGs regarding gene context.** Distribution of differential CpGs (FDR < 0.01; beta-value differential > 10%) among the different genomic locations around genes (3'UTR, body, first exon, 5'UTR, TSS200, TSS1500) and outside genes (intergenic). TSS200 and TSS1500 represent areas located at 200 bp and 1500 bp of transcription start sites, respectively. There is no significant bias of RS differential CpGs regarding gene context, whether against CLL or DLBCL. CpGs hypomethylated in RS vs CLL were enriched 1.5-fold in intergenic CpGs. CLL: chronic lymphocytic leukemia; DLBCL: *de novo* diffuse large B-cell lymphoma; FDR: false discovery rate; Hyper: hypermethylated; Hypo: hypomethylated; RS: Richter syndrome; TSS: transcription start site; UTR: untranslated region.

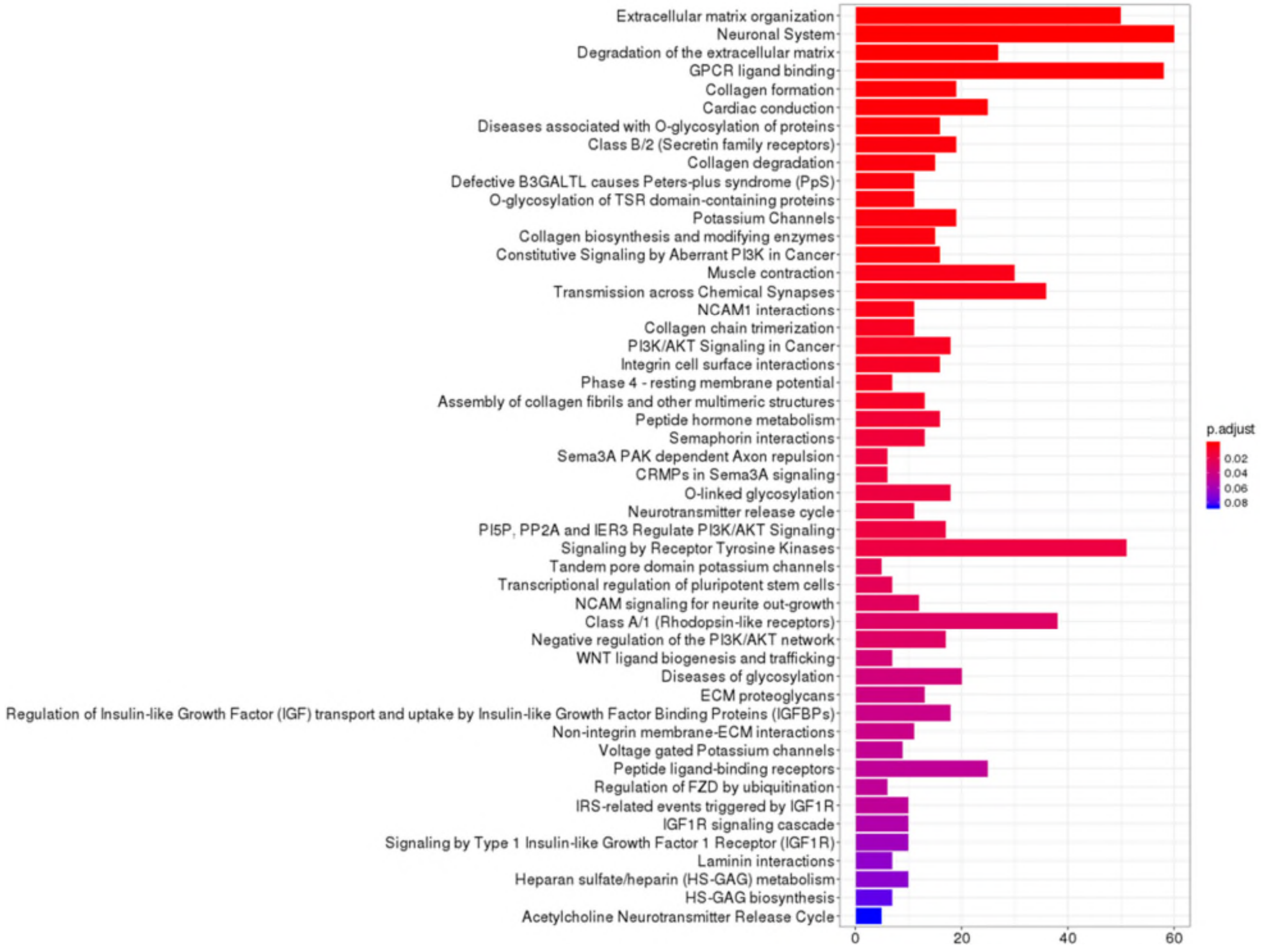


**Supplementary Fig. 9. RS vs DLBCL top functional annotations, as returned by ReactomePA.** Gene Set Enrichment Analysis permutation tests adjusted for the FDR. From 1,615 differential DMRs associated with 1,768 genes (min_smoothed_FDR, HMFDR and Fisher all < 0.01; max(beta-value differential) > 30%; at least 3 CpGs with gap < 1 kb). 98.9% of these DMRs were hypomethylated in RS. DLBCL: *de novo* diffuse large B-cell lymphoma; FDR: false discovery rate; Fisher: Fisher's multiple comparison statistic; HMFDR: harmonic mean of the individual components FDR; RS: Richter syndrome.

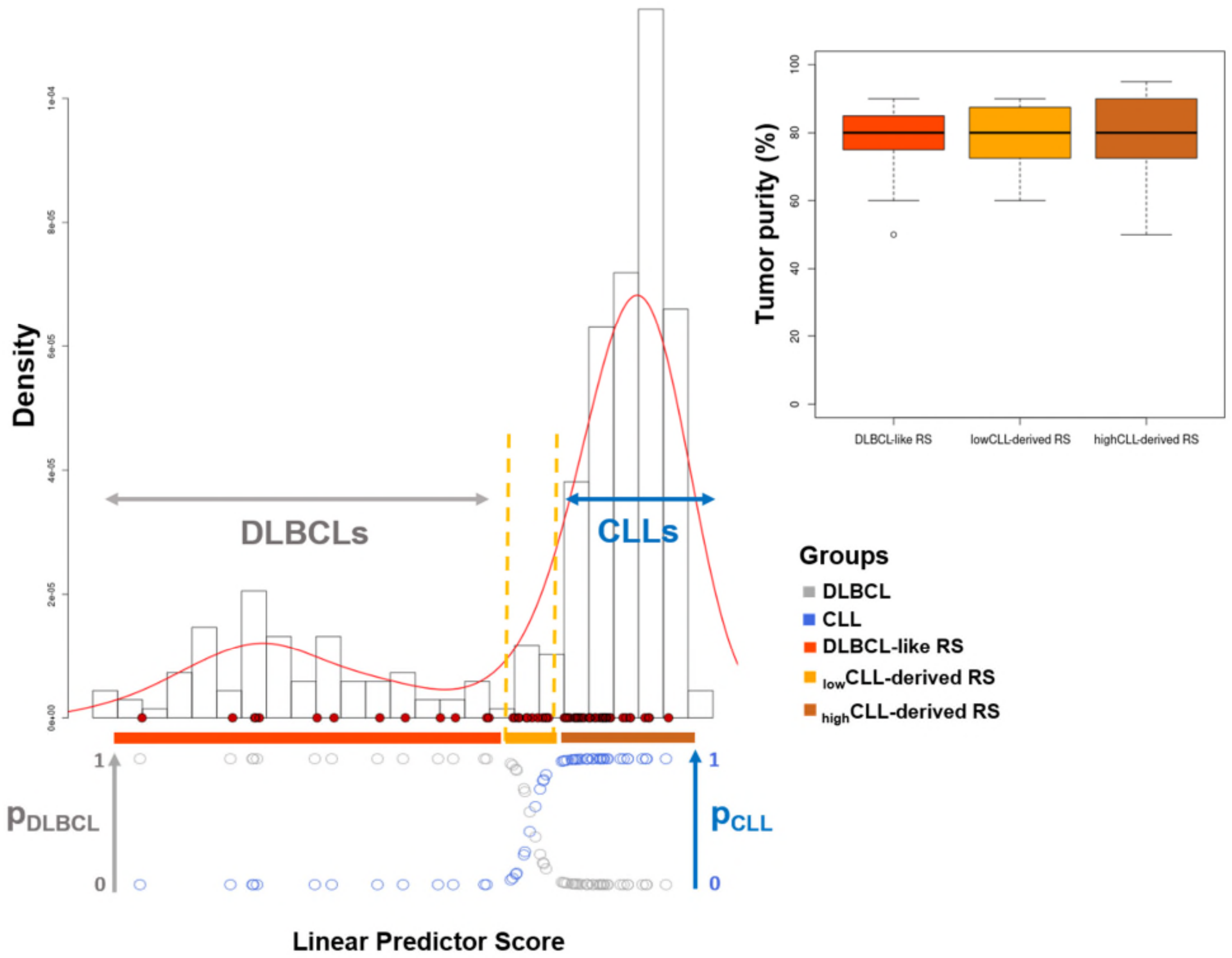


**Supplementary Fig. 10. Demonstration of normal distribution of linear predictor scores (LPS) within subgroups.** RS samples are displayed as red dots on the x-axis. Rationale for the 0.98 probability cut-off is found in the « unclassified zone » where the two normal distributions cross, between the dashed lines. Construction of the LPS is based on CLL vs DLBCL most differential CpGs, from which the IGHV signature has been subtracted. In grey and blue dots: application of the LPS on RS samples. Additionally, sample distribution is not a consequence of tumor purity (boxplots at the top-right side). Box plot: the center line, box limits, whiskers and points represent the mean, 25$^{th}$ and 75$^{th}$ percentile, 1.5x interquartile range and individual samples beyond the 1.5x interquartile, respectively. DLBCL-like RS group: n=13 independent samples; $_{low}$CLL-derived RS group: n=11 independent samples; $_{high}$CLL-derived RS group: n=32 independent samples. Source data are provided as a Source Data file. CLL: chronic lymphocytic leukemia; DLBCL: *de novo* diffuse large B-cell lymphoma; RS: Richter syndrome.

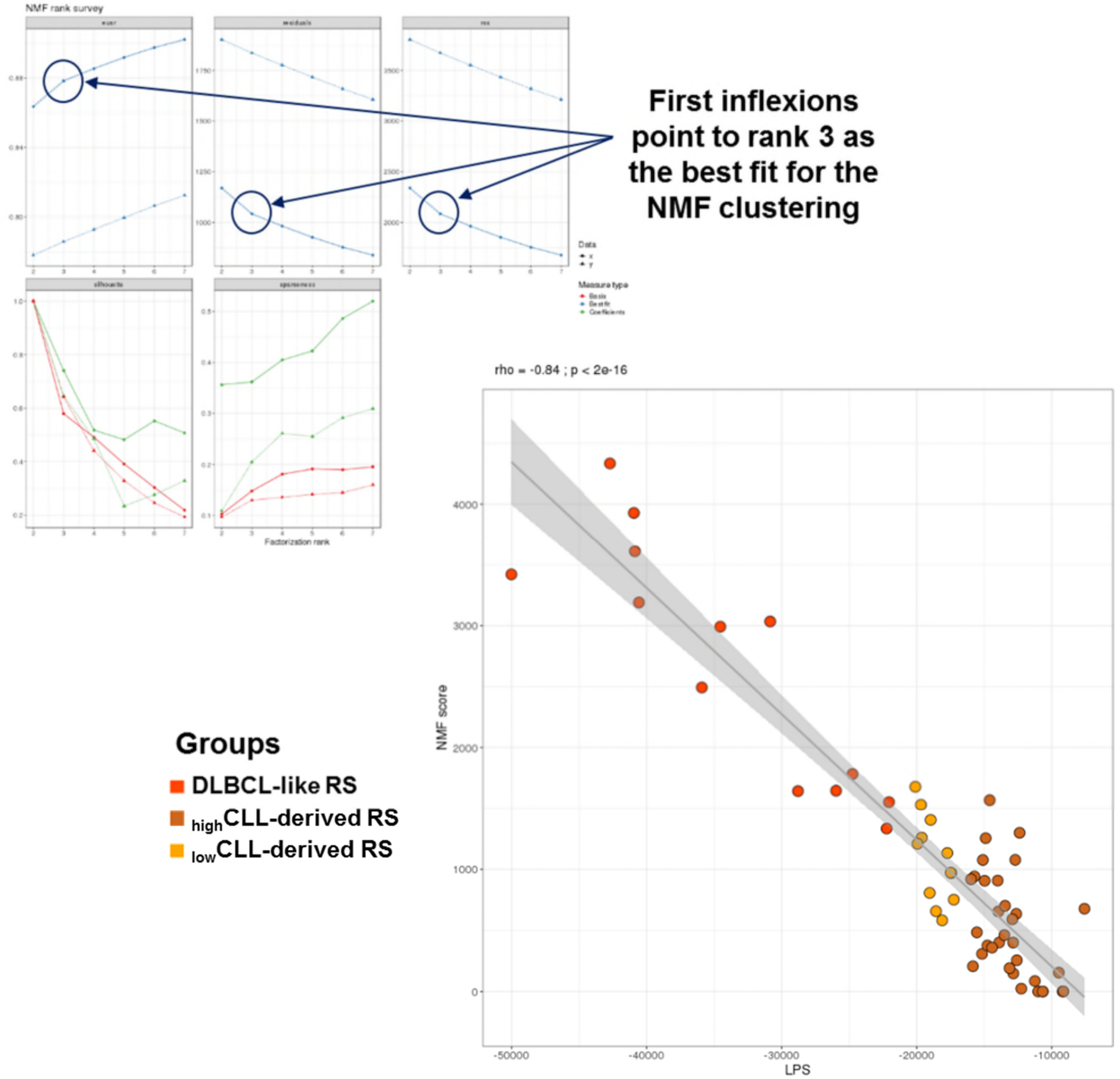


**Supplementary Fig. 11. Non-negative matrix factorization (NMF) classification of RS samples correlates with grouping by DNA methylome LPS.** Best sample classification is in 3 subgroups: rank 3 is the best fit as shown on the NMF rank survey (circles: test data; squares: best fit with test data; triangles: randomized data). On the figure, NMF clustering was performed with the 10,000 most differential CpGs from the EPIC dataset. Similar results have independently been obtained on every dataset considered (whether EPIC with 794,927 CpGs or FULL with 397,769 CpGs), with every CpGs, with the 10,000 most variant CpGs, or with the 4,863 LPS scoring CpGs (construction = CLLs + DLBCLs, test = RS). DLBCL-like RS: n=13; $_{low}$CLL-derived RS: n=12; $_{high}$CLL-derived RS: n=33; rho=-0.84; p<2e-16 (Spearman's correlation test). CLL: chronic lymphocytic leukemia; DLBCL: *de novo* diffuse large B-cell lymphoma; LPS: linear predictor score; NMF: non-negative matrix factorization; RS: Richter syndrome.

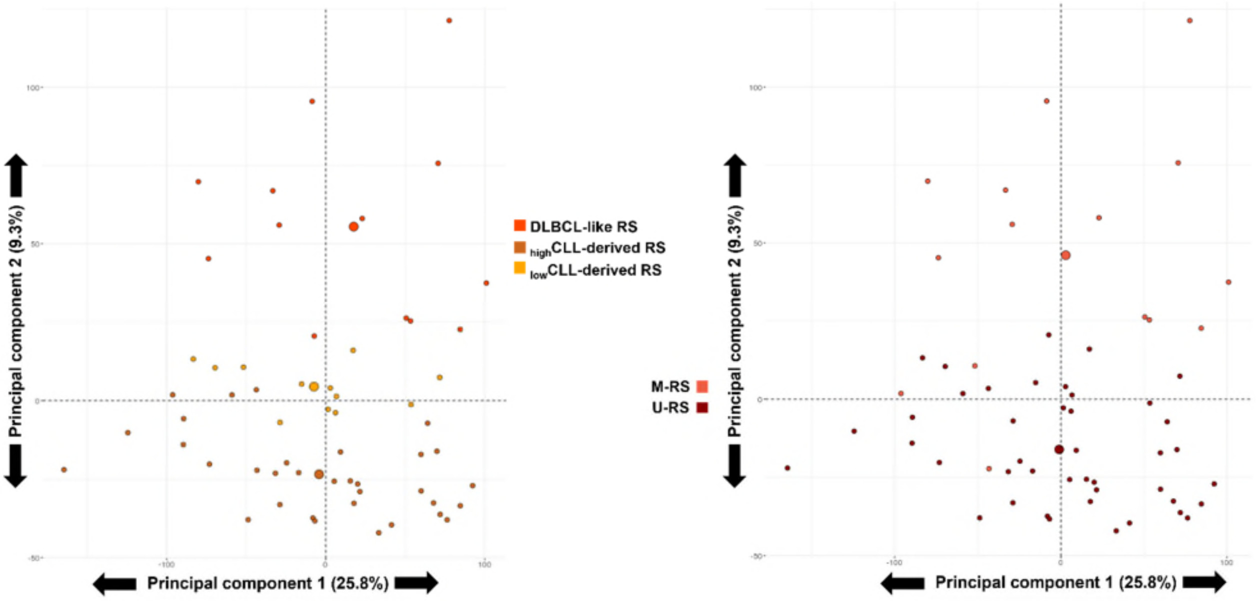


**Supplementary Fig. 12. Unsupervised PCA of all RS samples using the 794,927 CpGs from the EPIC dataset.** For each group, geometrical centers are displayed in bigger dots. Left panel: with RS subgroup annotations; Right panel: with *IGHV* mutational status. CLL: chronic lymphocytic leukemia; DLBCL: *de novo* diffuse large B-cell lymphoma; M-RS: IGHV-mutated RS; PCA: principal component analysis; RS: Richter syndrome; U-RS: IGHV-unmutated RS.

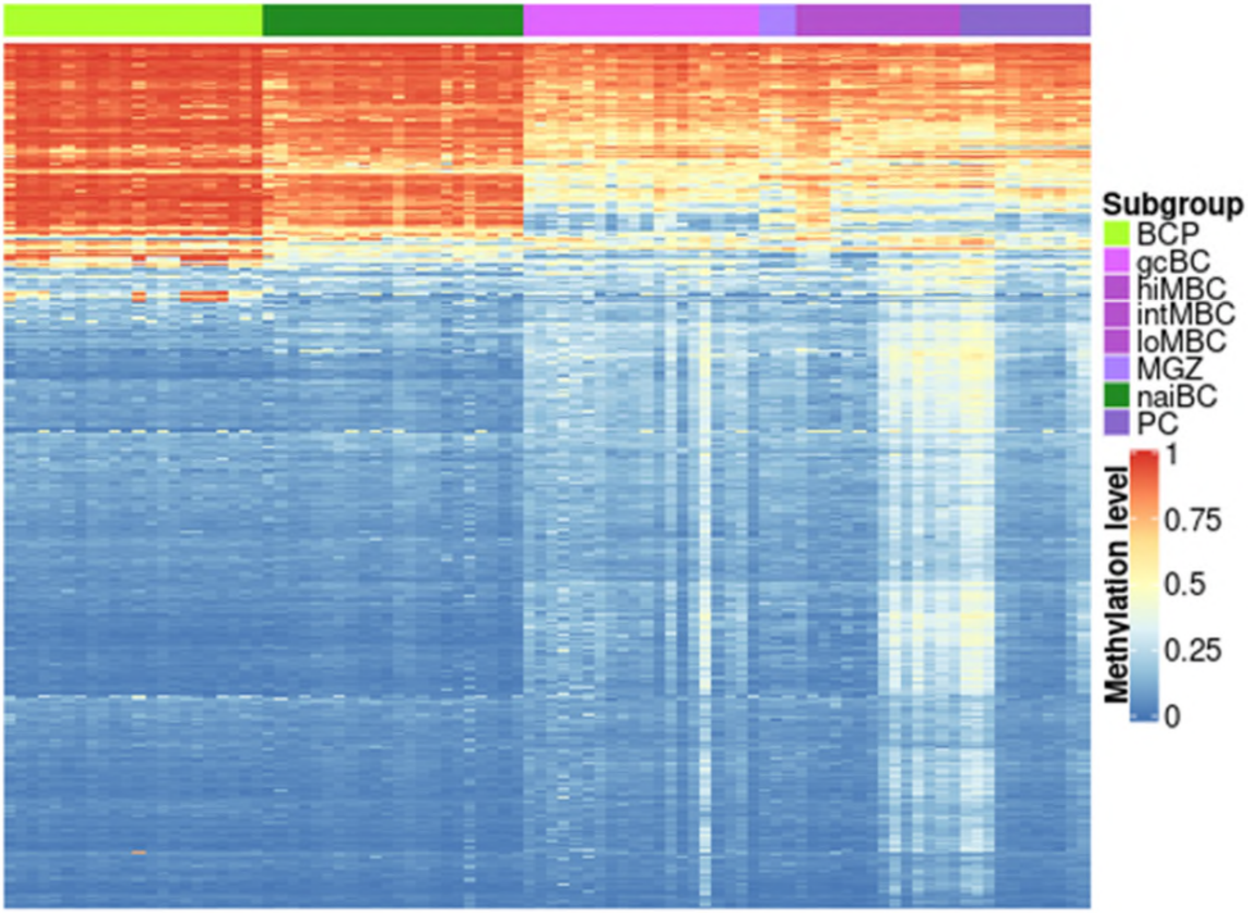


**Supplementary Fig. 13. Methylation status of the 4,863 CpGs used by the linear classifier score (LCS) in normal B-cells.** BCP: B-Cell precursors; gcBC: germinal center B-cells; MBC: memory B-cells; MGZ: marginal zone; naiBC: naive B-cells; PC: plasma cells.

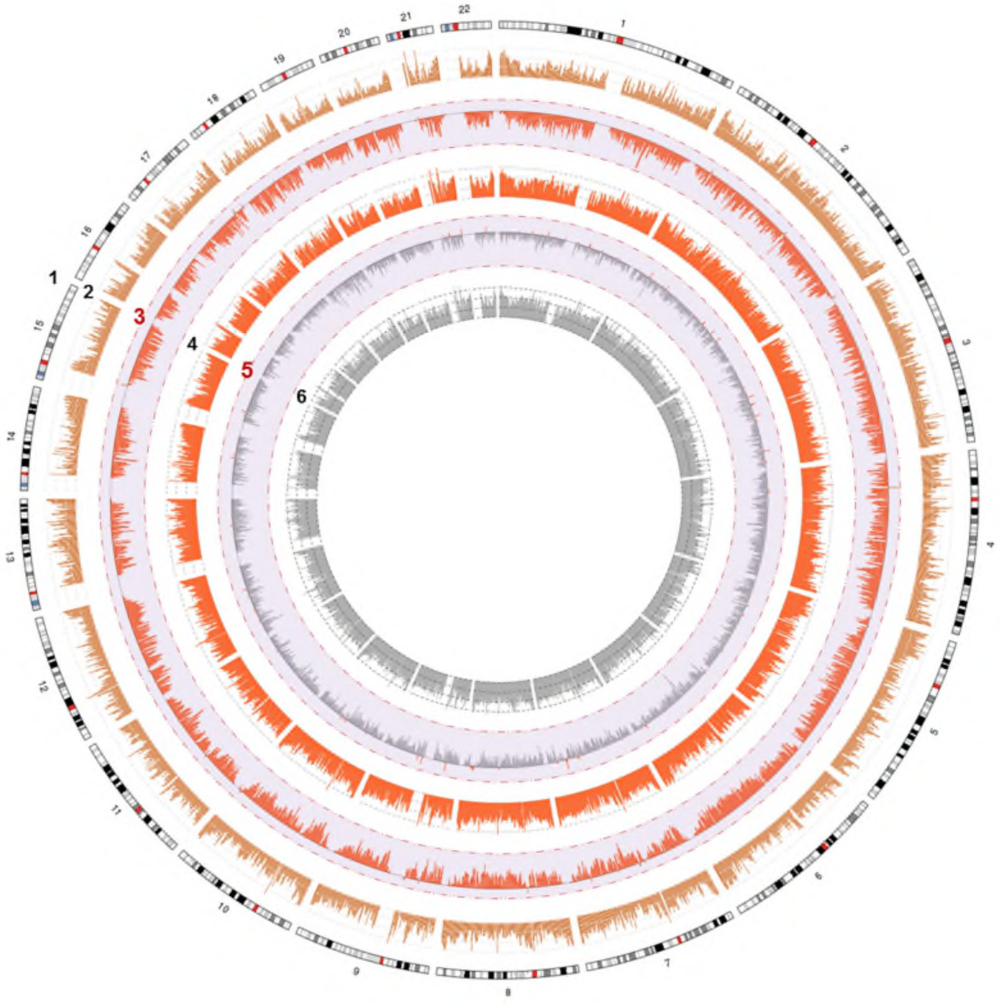


**Supplementary Fig. 14. Genomic distribution of DNA methylation in $_{high}$CLL-derived RS (brown), DLBCL-like RS (orange) and DLBCLs (grey).** Circular plot of median methylation levels and differences over sliding windows of 500 kb. Highlighted tracks 3 and 5 show the DNAm changes between $_{high}$CLL-derived, DLBCL-like RS and DLBCL, which are different in both extent and locations. From outer to inner track: 1) Chromosome number and ideograms with cytobands (X and Y chromosomes were removed from the analysis); 2) $_{high}$CLL-derived RS methylation levels; 3) $_{high}$CLL-derived minus DLBCL-like RS methylation differences (range -10 to +30%). Areas hypermethylated in DLBCL-like RS are represented with orange peaks pointing towards the DLBCL-like RS track; 4) DLBCL-like RS methylation levels; 5) DLBCL-like RS minus DLBCL methylation differences (range -19 to +7%). Areas hypermethylated in DLBCL-like RS are represented with orange peaks pointing toward the DLBCL-like RS track while areas hypermethylated in DLBCLs are represented with grey peaks pointing towards the DLBCL track; 6) DLBCL methylation levels. Dashed lines represent 0%, 25%, 50%, and 75% average methylation. CLL: chronic lymphocytic leukemia; DLBCL: *de novo* diffuse large B-cell lymphoma; RS: Richter syndrome.

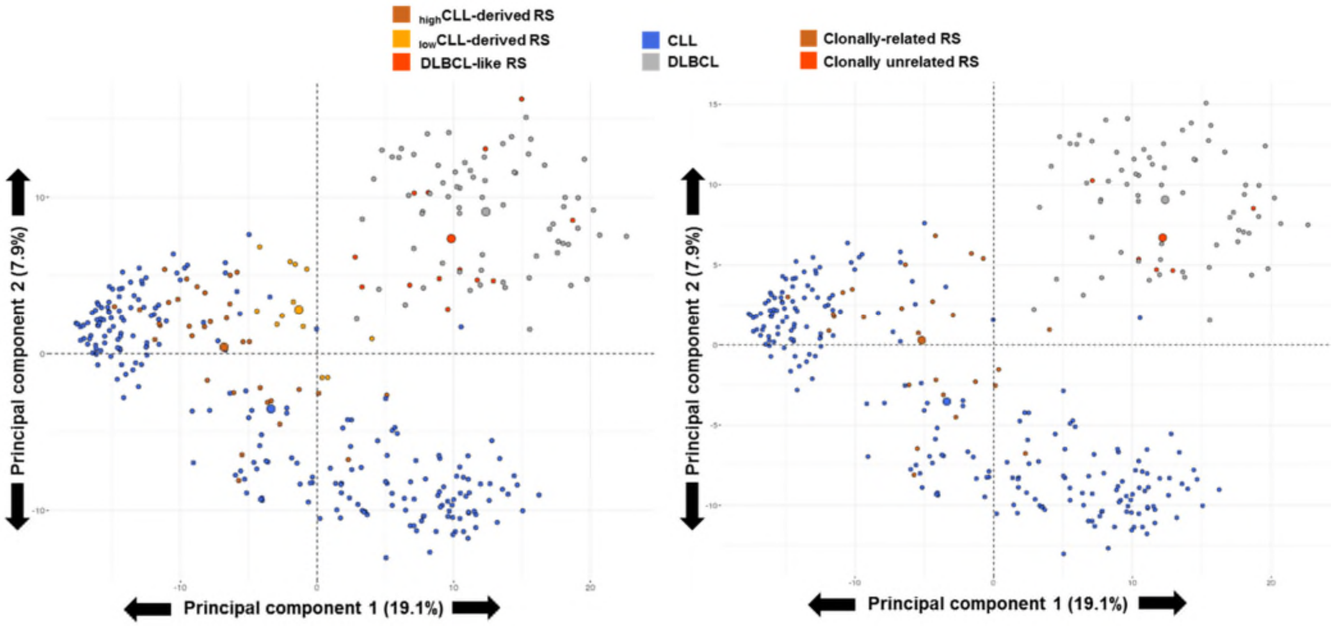


**Supplementary Fig. 15. Sample partitioning according to IGHV mutational status.** Additional data for Figure 3C, PCA on the 10,000 most variable CpGs of the dataset. Left panel: samples are tagged according to DNA methylation profile; Right panel: samples are tagged according to CLL-RS clonal relationship. RS samples with unknown clonality status are hidden. For each group, geometrical centers are displayed in bigger dots. CLL: chronic lymphocytic leukemia; DLBCL: *de novo* diffuse large B-cell lymphoma; PCA: principal component analysis; RS: Richter syndrome.

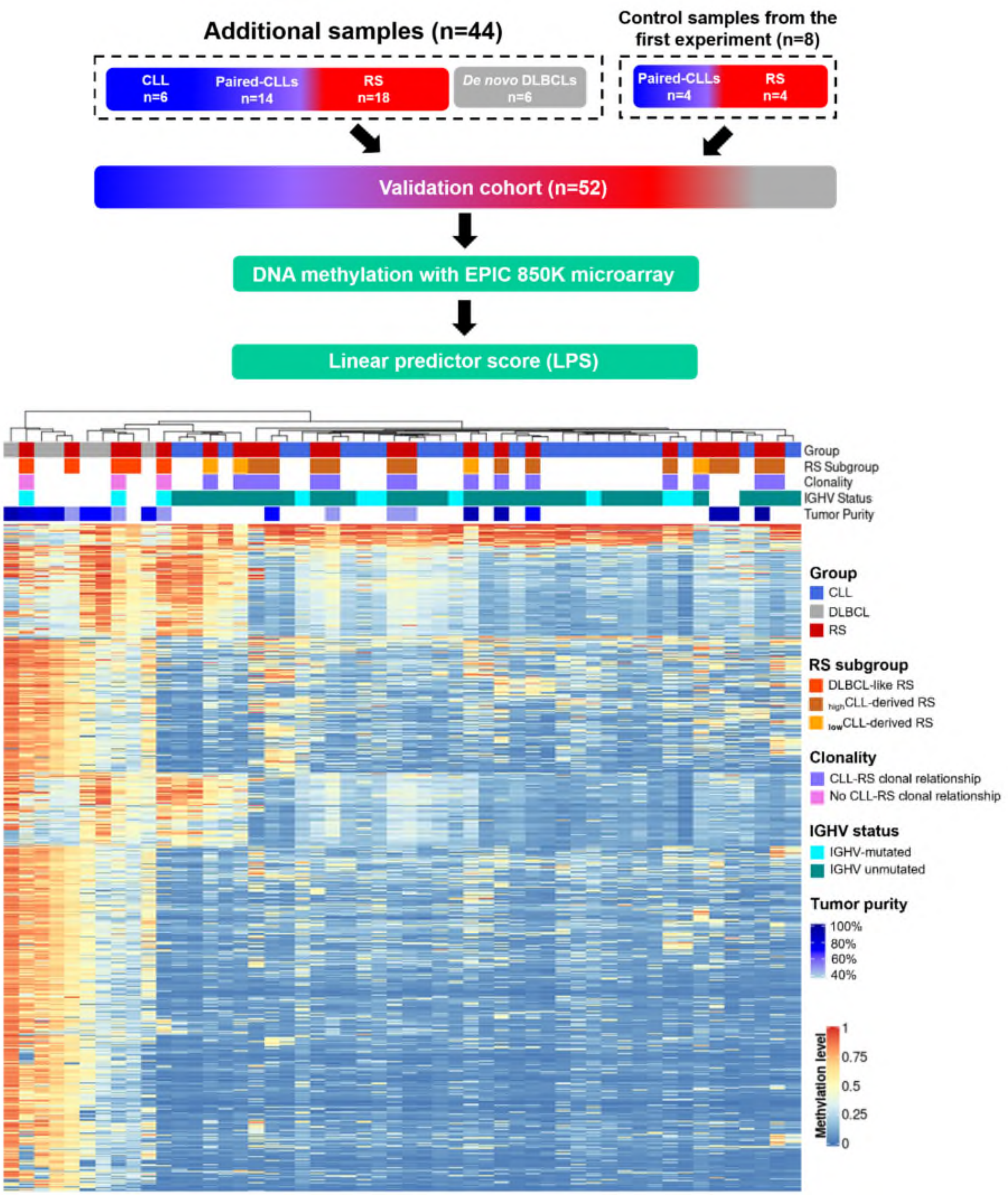


**Supplementary Fig. 16. Hierarchical clustering of DNAm data retrieved from the validation cohort.** This 52-sample validation cohort included 44 new samples: 18 new RS samples, the CLL component of 14 of these, 6 new DLBCL samples, and 6 additional CLLs. In addition, 8 samples from the training series were used as controls: 4 RS samples (3 clonally related and 1 clonally unrelated), with the 4 respective CLL components. RS sample classification according to LPS is displayed in the "RS Subgroup" annotations. CLL: chronic lymphocytic leukemia; DLBCL: *de novo* diffuse large B-cell lymphoma; LPS: linear predictor score; RS: Richter syndrome.

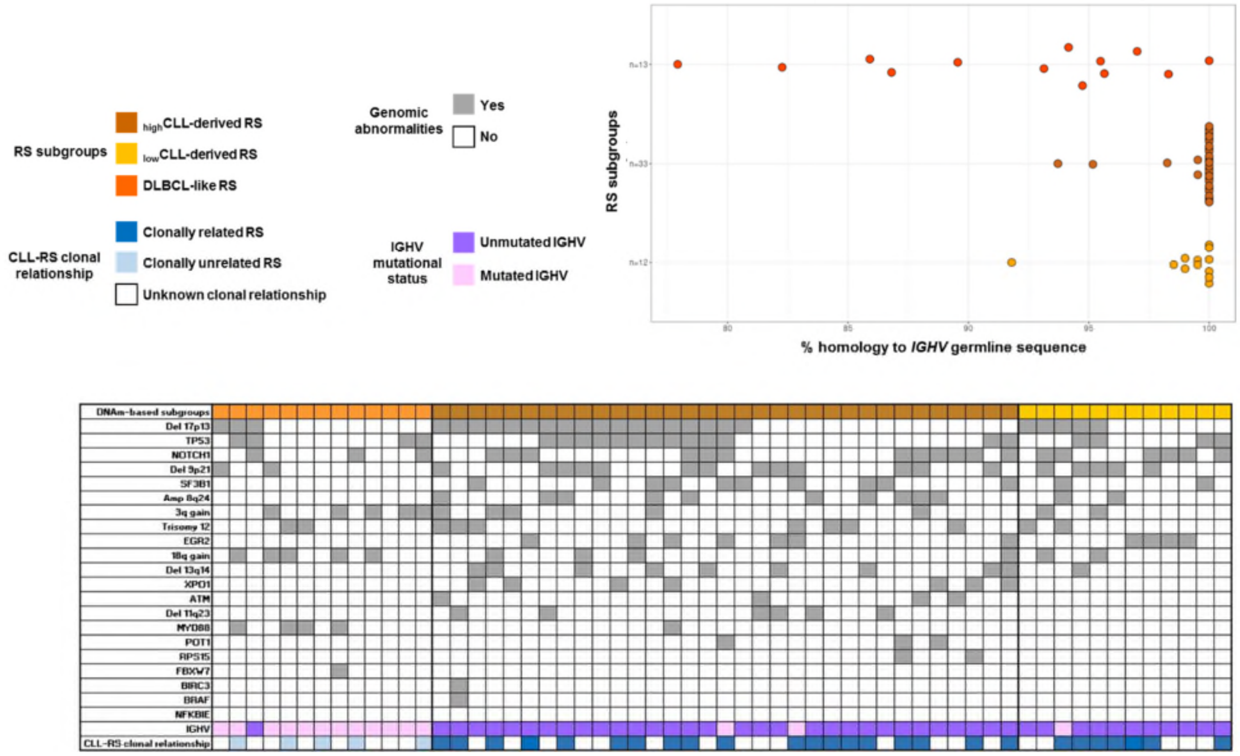


**Supplementary Fig. 17. Annotation of RS DNA methylation subgrouping with a panel of genomic abnormalities frequently described in CLL and RS.** CLL: chronic lymphocytic leukemia; RS: Richter syndrome.

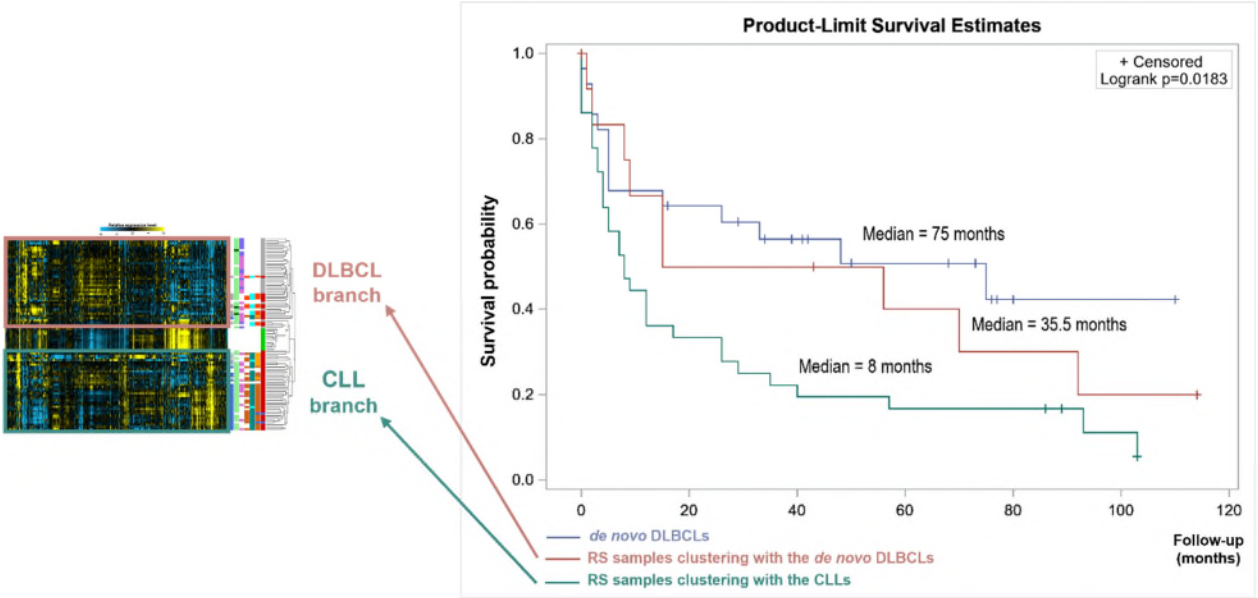


**Supplementary Fig. 18. CLL-derived RS are associated with a worse prognosis than DLBCL-like RS and *de novo* DLBCL.** Kaplan-Meier estimates of overall survival for 77 patients. Statistical comparisons were performed with Log-rank test. Bonferroni method was used for multitesting adjustment. CLL: chronic lymphocytic leukemia; DLBCL: diffuse large B-cell lymphoma; RS: Richter syndrome.

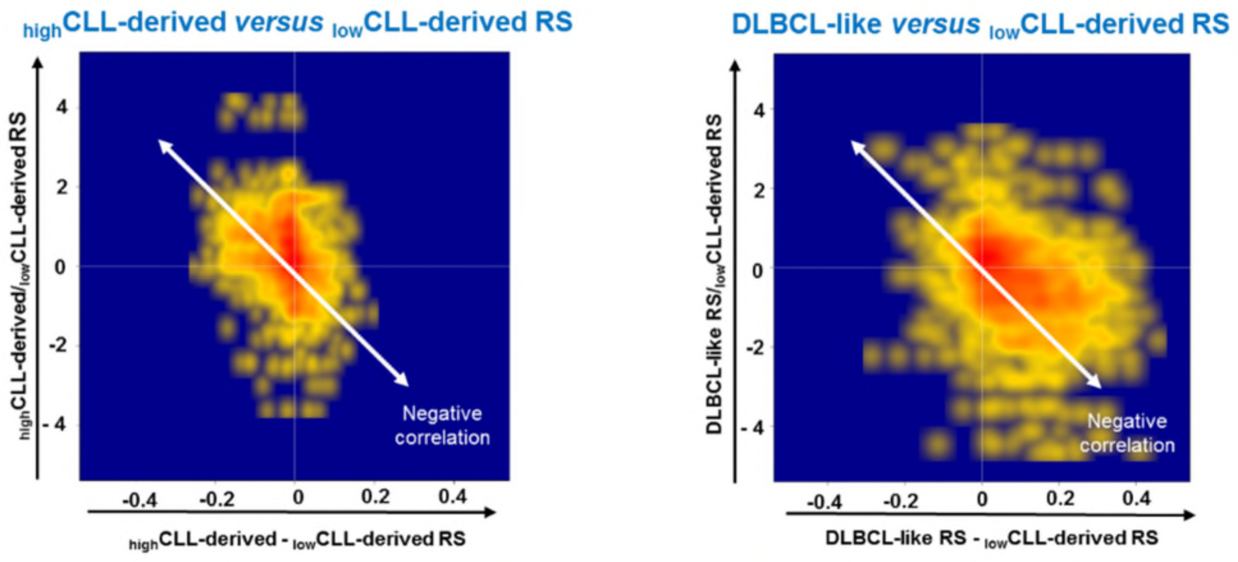


**Supplementary Fig. 19. Density maps (smoothed density scatterplots) representing overall DNA methylation vs gene expression changes.** Scale ranges from blue (no density), to yellow (medium density) and red (high density). Only genes with at least 1 significant correlation (cut-off p-value < 0.01) were retained. The exact same limits for x and y axes are chosen for each plot. CLL: chronic lymphocytic leukemia; DLBCL: *de novo* diffuse large B-cell lymphoma; RS: Richter syndrome.

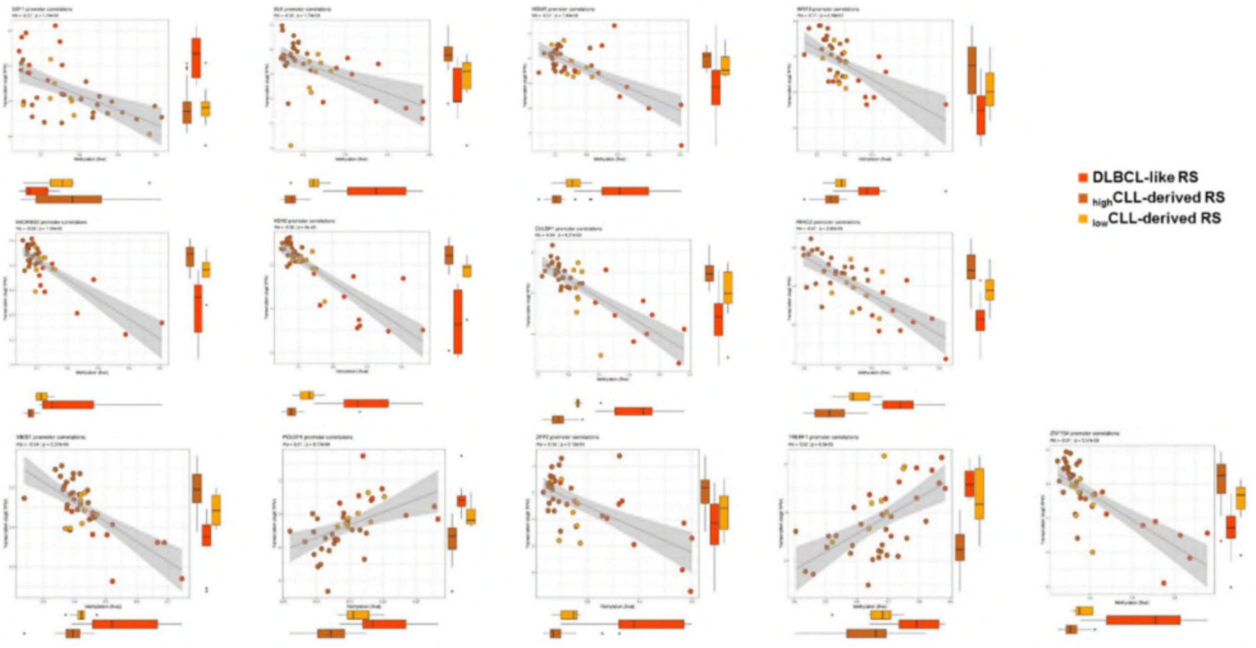


**Supplementary Fig. 20. Correlation scatterplots between DNA methylation and gene expression values, viewed for a selection of genes. X**-axis: DNA methylation average beta-value for negatively or positively correlating regulatory regions (see main text and Methods); y-axis: transcriptional activity of the corresponding gene, in transcript per million (TPM, normalized expression). Boxplots are displayed at the bottom and on the right hand side for methylation and transcription summaries, respectively. DLBCL-like RS group: n=9 independent samples; $_{low}$CLL-derived RS group: n=8 independent samples; $_{high}$CLL-derived RS group: n=24 independent samples. For all box plots, the center line, box limits, whiskers and points represent the median, $25^{th}$ and $75^{th}$ percentile, 1.5x interquartile range and individual samples beyond the 1.5x interquartile, respectively. Bval: Beta-value; CLL: chronic lymphocytic leukemia; DLBCL: *de novo* diffuse large B-cell lymphoma; RS: Richter syndrome; TPM: transcript per million.

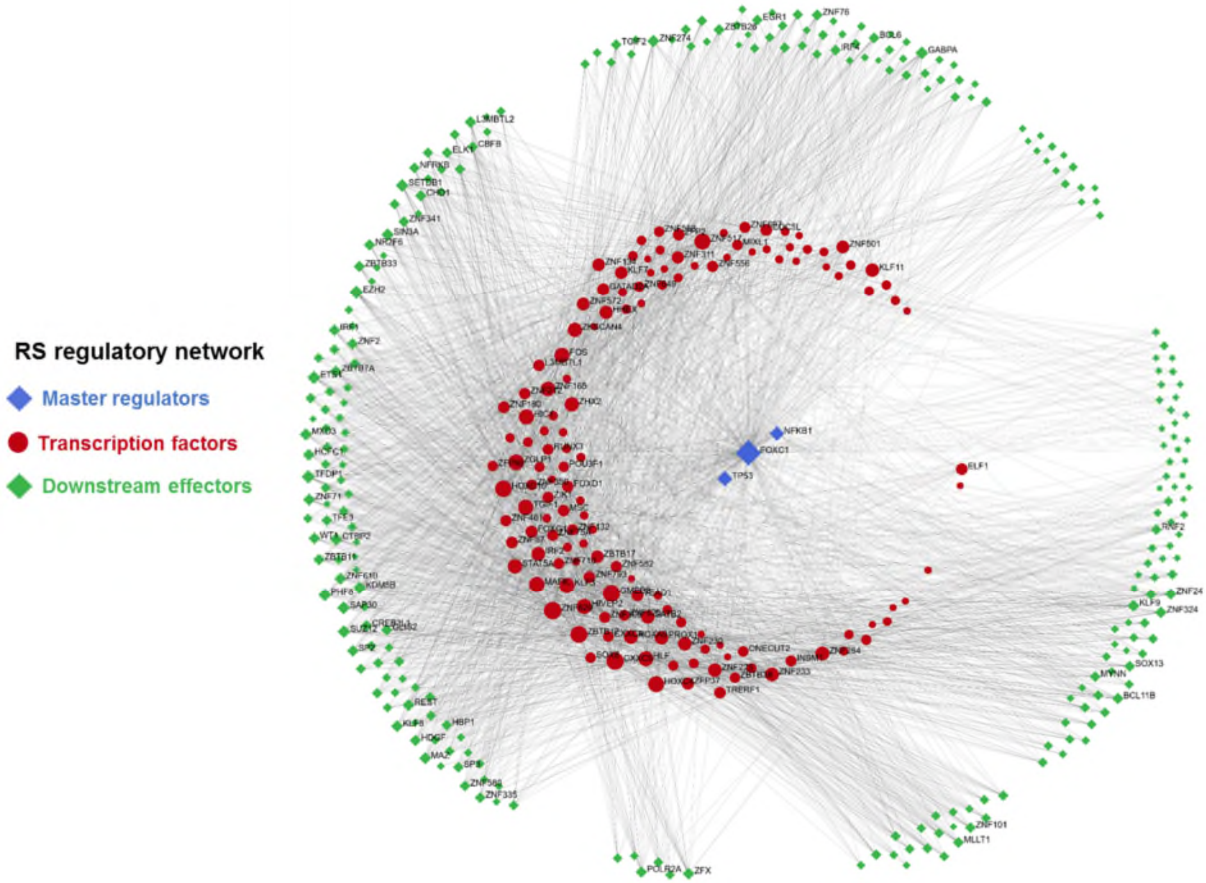


**Supplementary Fig. 21. Regulatory network inferred from the set of 156 transcription factors correlating between methylome and transcriptome.** These TFs are used as seeds for reconstructing the network (red dots, 1st line). Upstream, they are controlled by three master regulators (TP53, NFKB1 and FOXC1, blue dots). In turn, they regulate a large set of downstream TF effectors (green dots; 2nd line). TF interactions were computed from ENCODE and JASPAR databases. TF: transcription factor.

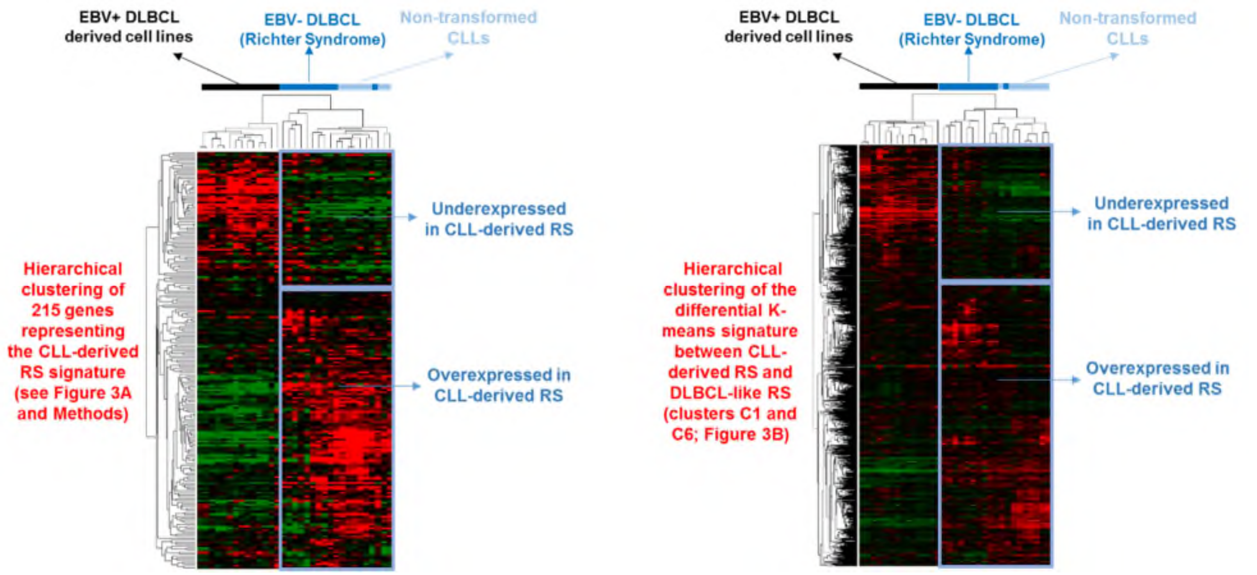


**Supplementary Fig. 22. Validation of the CLL-derived RS gene signature on a DLBCL cohort including RS samples.** From the public dataset GSE103265 available from the Gene Expression Omnibus database. CLL: chronic lymphocytic leukemia; DLBCL: diffuse large B-cell lymphoma; EBV: Epstein-Barr virus; RS: Richter syndrome.

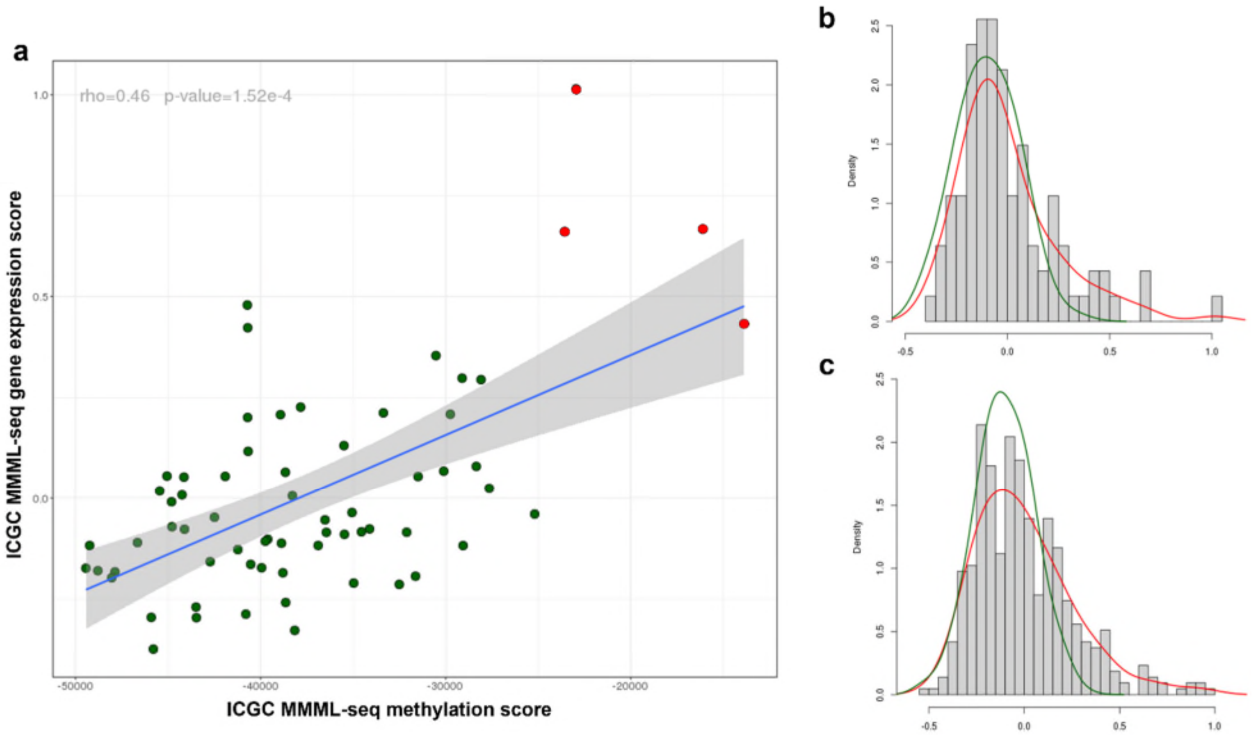


**Supplementary Fig. 23. a** Correlation between methylome and transcriptome scores (LPS and LCS, respectively; Spearman's test) in the ICGC MMML-seq consortium dataset (scatterplot, linear modelling fit with 5% confidence interval). Samples with extreme scores in both DNAm and gene expression are labeled in red; rho=0.46, p=1.52e-4.**b** LCS densities for the ICGC MMML-seq consortium dataset (n=94); **c)** LCS densities for MMML dataset (n=430). **b** and **c** Red curve: observed distribution (lowess-smoothed); Green curve: simulated/expected distribution from the real observations (lowess-smoothed). ICGC: International Cancer Genome Consortium; LCS: linear classifier score (from gene expression); LPS: linear predictor score (from DNA methylation); MMML: Molecular Mechanisms in Malignant Lymphoma.

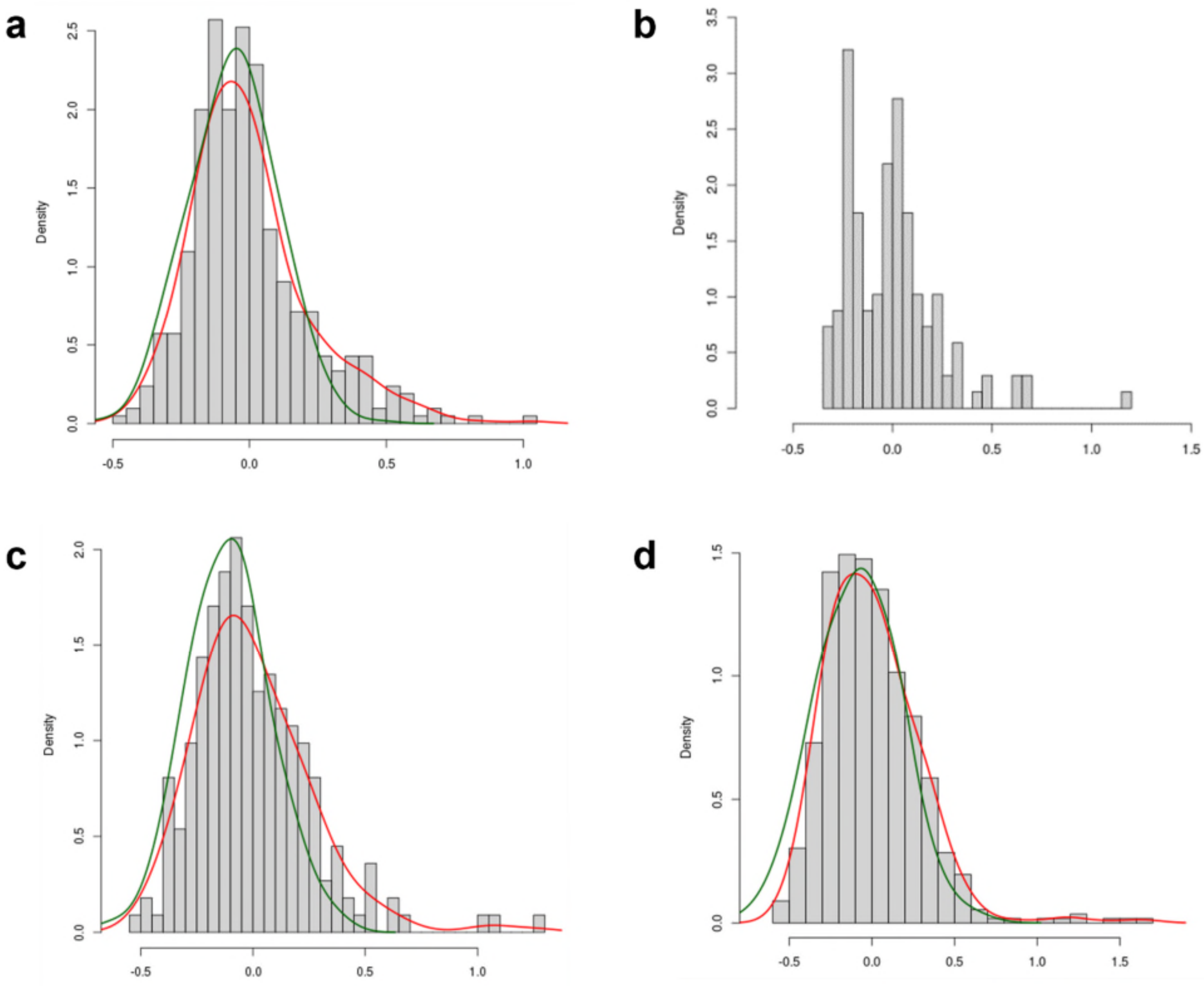


**Supplementary Fig. 24. Linear classifier score distributions for all other studied datasets.** Histograms of LCS from: **a** Lenz dataset (n=420; microarray, accession under GSE10846; PMID: 21546504); **b** Chapuy dataset (n=137; microarray, accession under GSE98588; PMID: 29713087); **c** Dubois dataset (n=223; microarray, accession under GSE87371; PMID: 31648986); **d** Wright dataset (n=562; RNA-Seq; PMID: 32289277). Red curve: observed distribution (lowess-smoothed); Green curve: simulated/expected distribution from the real observations (lowess-smoothed). LCS: linear classifier score.

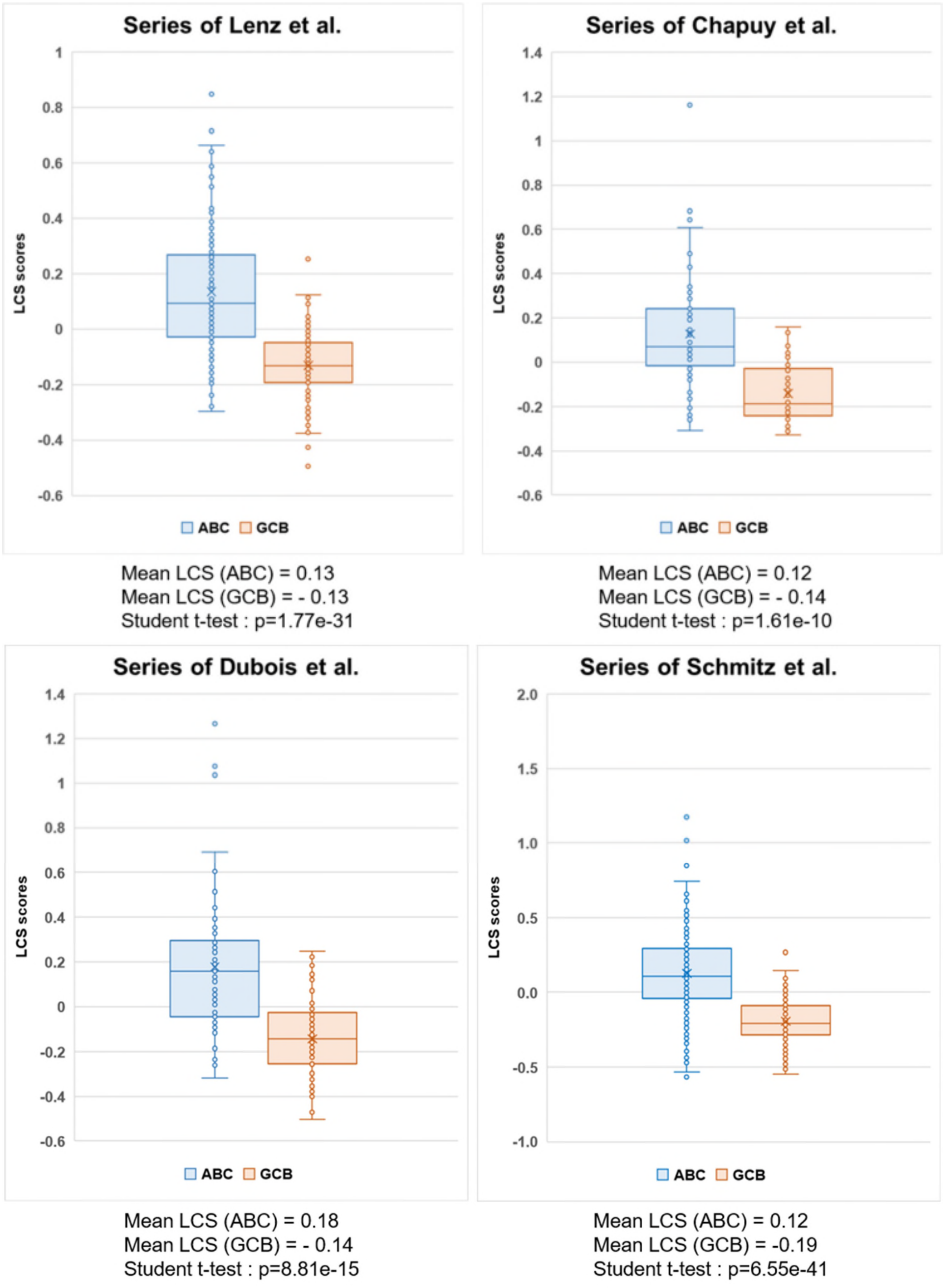


**Supplementary Fig. 25. Distribution of LCS scores among ABC- and GCB-subtype DLBCLs in 4 large series.** Series from Lenz et al: n=167 independent ABC subtype DLBCL cases and n= 183 independent GCB subtype DLBCL cases. Series from Chapuy et al: n=63 independent ABC subtype DLBCL cases and n=53 independent GCB subtype DLBCL cases. Series from Dubois et al: n=83 independent ABC subtype DLBCL cases and n=85 independent GCB subtype DLBCL cases. Series from Schmidt et al: n=286 independent ABC subtype DLBCL cases and n=162 independent GCB subtype DLBCL cases. Box plots: the center line, box limits, whiskers and points represent the median, 25th and 75th percentile, 1.5x interquartile range and individual samples, respectively. Two-sided Student's t-test. Source data are provided as a Source Data file. ABC: activated B-cell like; GCB: germinal center B-cell like; LCS: linear classifier score.

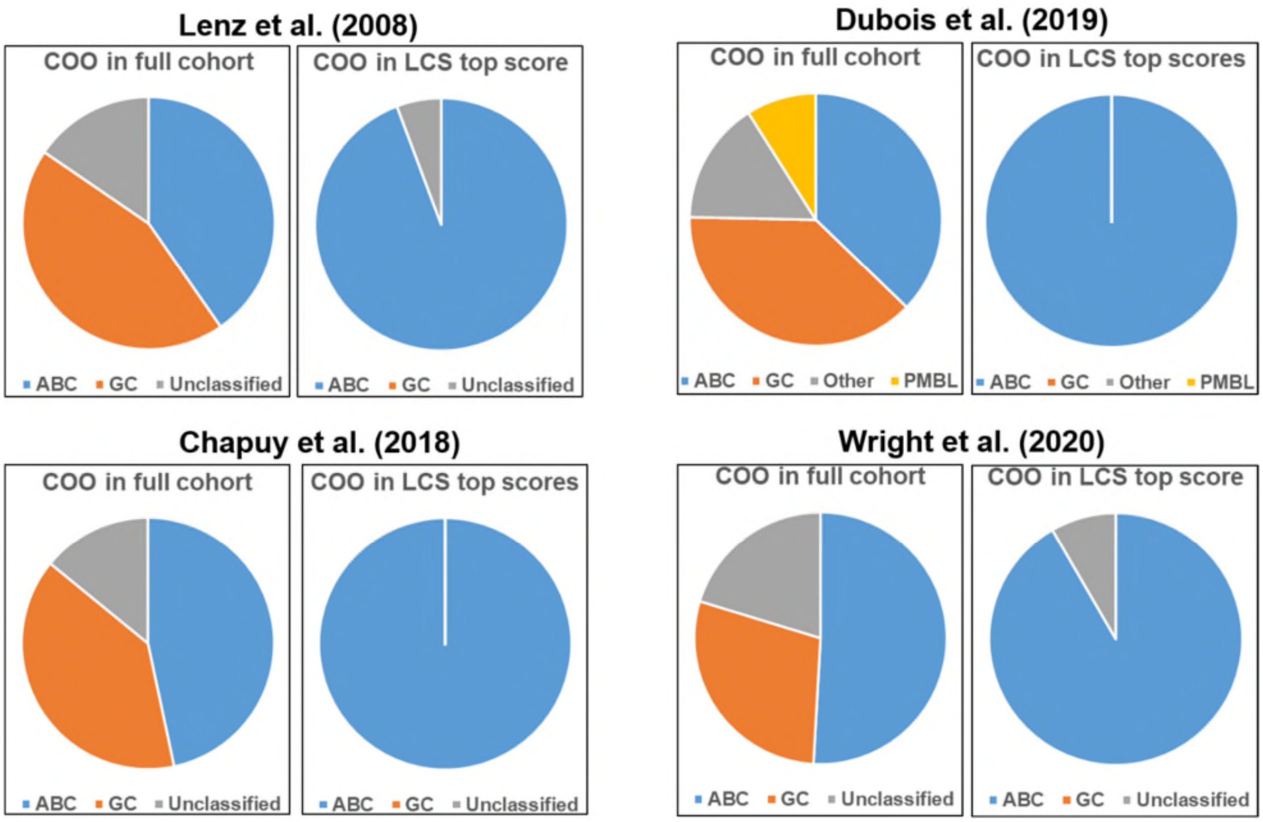


**Supplementary Fig. 26. Distribution of COO in the entire cohorts and among the top-LCS samples in 4 datasets.** ABC: activated B-cell like; COO: cell-of-origin; GCB: germinal center B-cell like; LCS: linear classifier score.

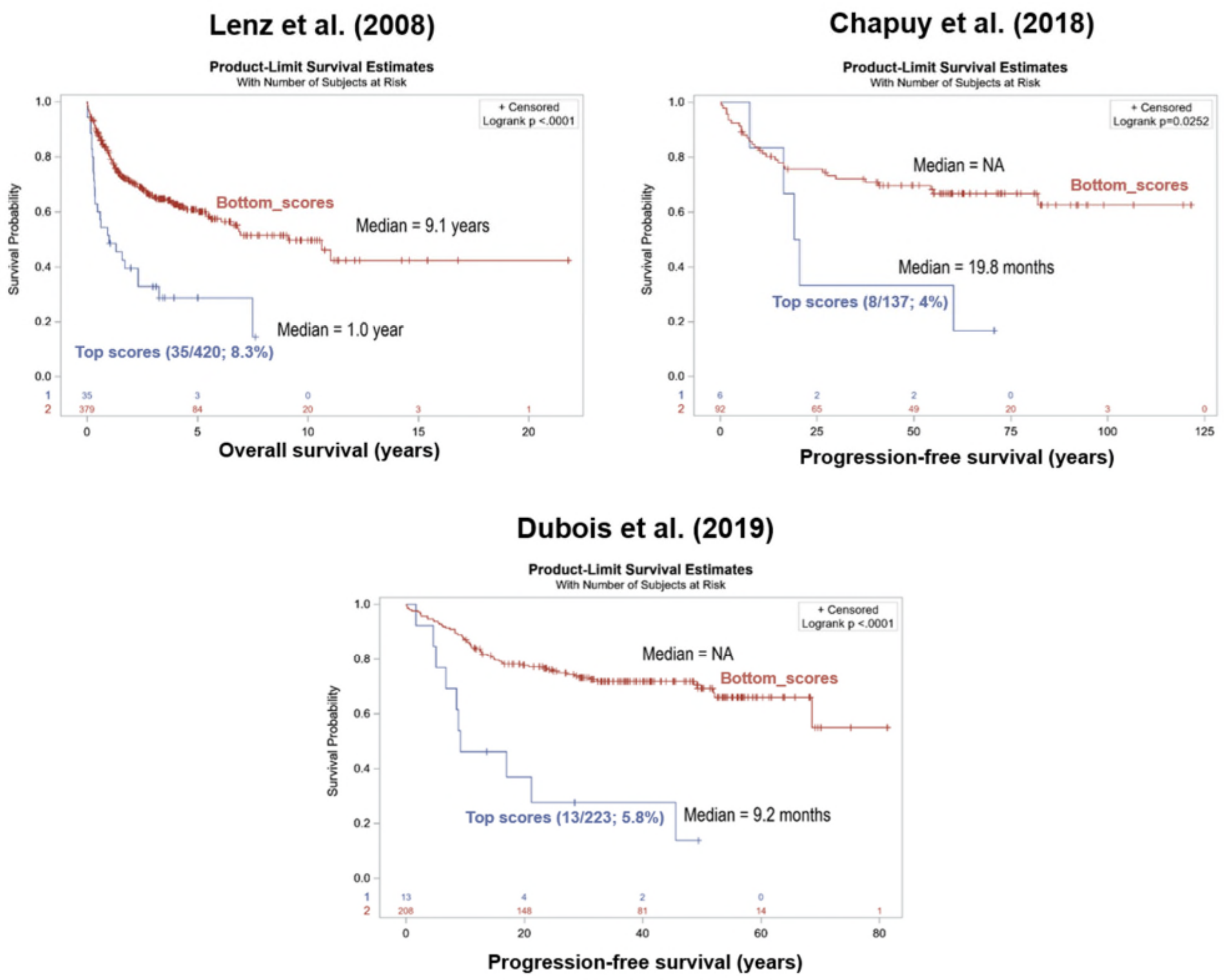


**Supplementary Fig. 27. Kaplan-Meier estimates of overall survival or progression-free survival for 733 patients from three DLBCL datasets comparing patients with top-LCS and the rest of the cohorts.** Series from Lenz and colleagues: n=420 patients; p=3.5e-7; series from Chapuy and colleagues: n=137 patients; p=0.0252; series from Dubois and colleagues: n=223 patients; p=3.2e-6. Statistical comparisons were performed with the log-rank test.

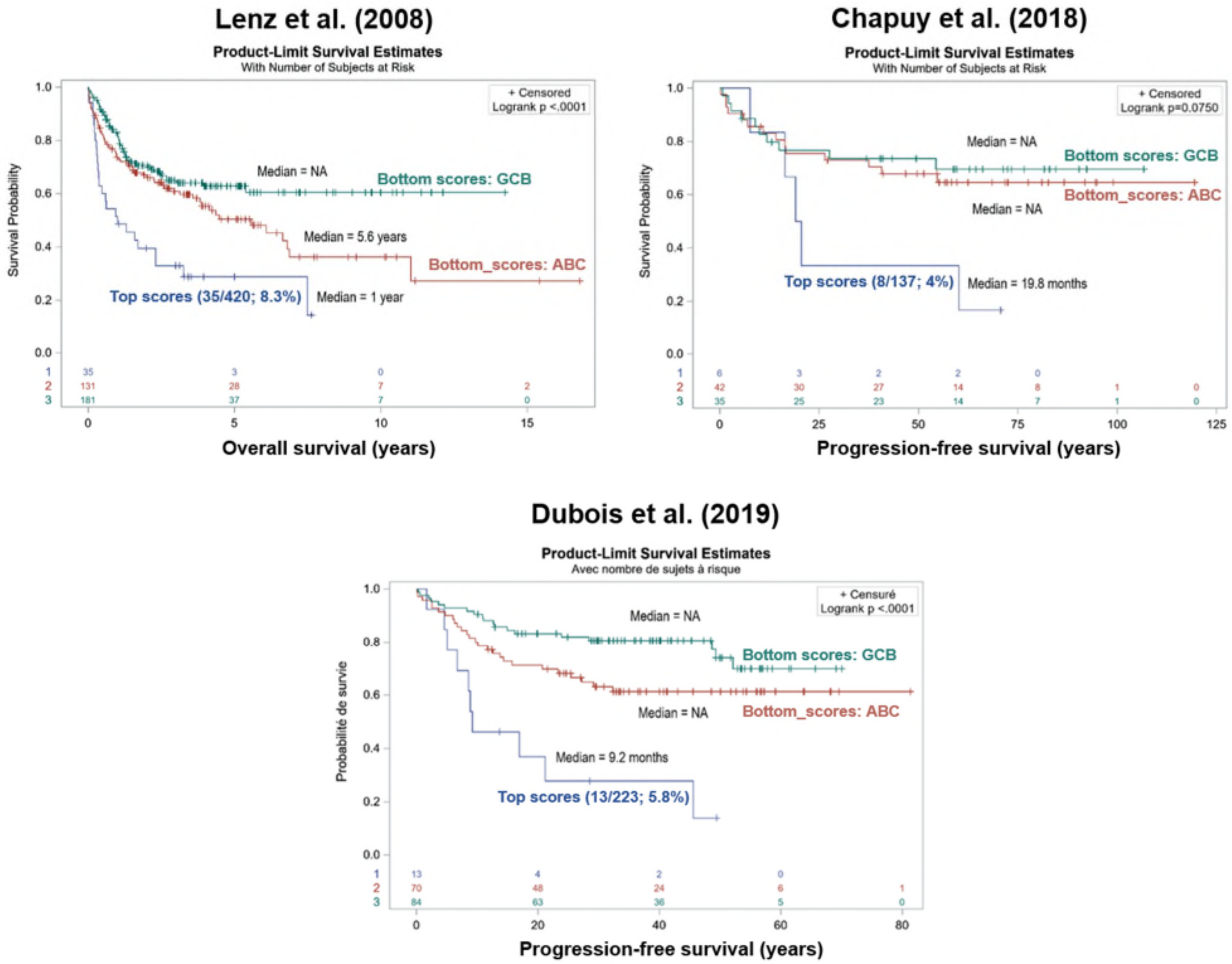


**Supplementary Fig. 28. Kaplan-Meier estimates of overall survival or progression-free survival for 733 patients from three DLBCL datasets.** Comparison between patients with top LCS and the rest of the cohorts, according to COO. Series from Lenz and colleagues: n=420 patients; p=5.2e-6; series from Chapuy and colleagues: n=137 patients; p=0.075; series from Dubois and colleagues: n=223 patients; p=4.2e-6. Statistical comparisons were performed with the log-rank test. Bonferroni method was used for multitesting adjustments. COO: cell-of-origin; DLBCL: *de novo* diffuse large B-cell lymphoma.

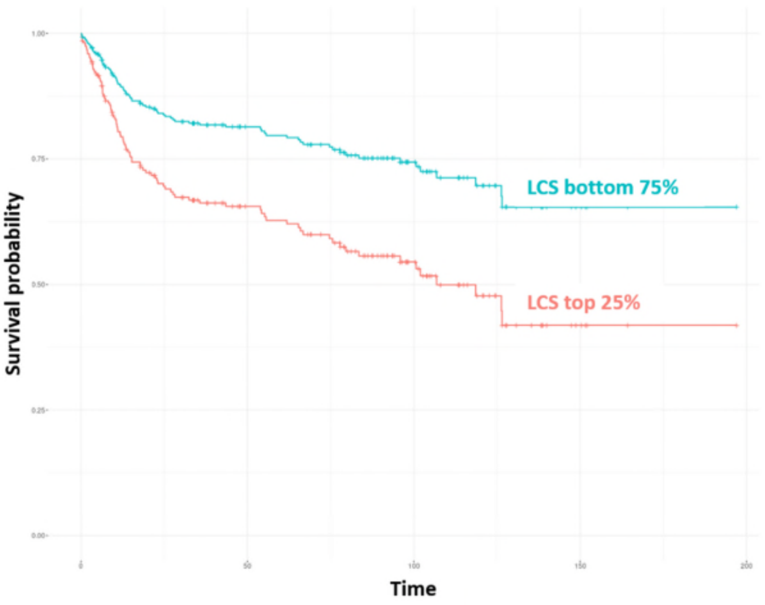


**Cox PH multivariate statistics for PFS, variable = LCS, covariates = IPI + TP53 + DoubleHit (LR, Wald & logrank tests all < 1e-7)**

| | Beta | HR | P-Value | 95% CI [LL, UL] | |
|---|---|---|---|---|---|
| LCS | 0,4421 | 1,556 | 8,16E-05 | [1.2488,1.939] | *** |
| IPIcategory | 0,7628 | 2,1442 | 2,11E-06 | [1.5645,2.939] | *** |
| TP53 | 0,41 | 1,5068 | 0,275 | [0.7218,3.146] | |
| DoubleHit | -0,19 | 0,8269 | 0,569 | [0.4296,1.592] | |

**Cox PH multivariate statistics for OS, variable = LCS, covariates = IPI + TP53 + DoubleHit (LR, Wald & logrank tests all < 9e-7)**

| | Beta | HR | P-Value | 95% CI [LL, UL] | |
|---|---|---|---|---|---|
| LCS | 0,36538 | 1,44106 | 0,00338 | [1.1287,1.84] | ** |
| IPIcategory | 0,8426 | 2,32241 | 9,26E-07 | [1.6587,3.252] | *** |
| TP53 | 0,01682 | 1,01696 | 0,96862 | [0.44,2.351] | |
| DoubleHit | -0,23064 | 0,79402 | 0,52805 | [0.3879,1.625] | |

**Cox PH multivariate statistics for PFS, variable = top25, covariates = IPI + TP53 + DoubleHit (LR, Wald & logrank tests all < 2e-6)**

| | Beta | HR | P-Value | 95% CI [LL, UL] | |
|---|---|---|---|---|---|
| top25 | 0,7127 | 2,0395 | 0,00327 | [1.2683,3.279] | ** |
| IPIcategory | 0,7805 | 2,1826 | 8,68E-07 | [1.5993,2.979] | *** |
| TP53 | 0,3648 | 1,4402 | 0,33169 | [0.6895,3.008] | |
| DoubleHit | -0,1739 | 0,8404 | 0,59978 | [0.4389,1.609] | |

**Cox PH multivariate statistics for OS, variable = top25, covariates = IPI + TP53 + DoubleHit (LR, Wald & logrank tests all < 5e-6)**

| | Beta | HR | P-Value | 95% CI [LL, UL] | |
|---|---|---|---|---|---|
| top25 | 0,587363 | 1,799238 | 0,0265 | [1.0708,3.023] | * |
| IPIcategory | 0,861689 | 2,367155 | 4,48E-07 | [1.694,3.308] | *** |
| TP53 | 0,007843 | 1,007874 | 0,9854 | [0.4353,2.334] | |
| DoubleHit | -0,212922 | 0,808219 | 0,5566 | [0.3974,1.644] | |

**Supplementary Fig. 29. Multivariate analysis with a Cox proportional hazards model, including all available informative covariates to evaluate the association of LCS with survival (OS and PFS).** This association was calculated in binary (top 25% LCS versus the rest, tagged LCS in the tables) as well as linear (LCS as a continuous variable, tagged Zscore in the table) modes. Cox proportional-hazards multivariate models: (i) PFS model includes LCS, IPI, *TP53* abnormalities, and *MYC/BCL2* double hit (Wald = 1e-8); (ii) OS model includes LCS, IPI, *TP53* abnormalities and *MYC/BCL2* double hit (Wald = 5e-7). Statistical tests (Wald, LR) are two-tailed. CI: confidence interval, HR: hazard ratio; LCS: linear classifier score; LL: lower limit; LR: likelihood ratio; UL: upper limit. *: p-value < 0.05; **: p-value < 0.01; ***: p-value < 0.001.

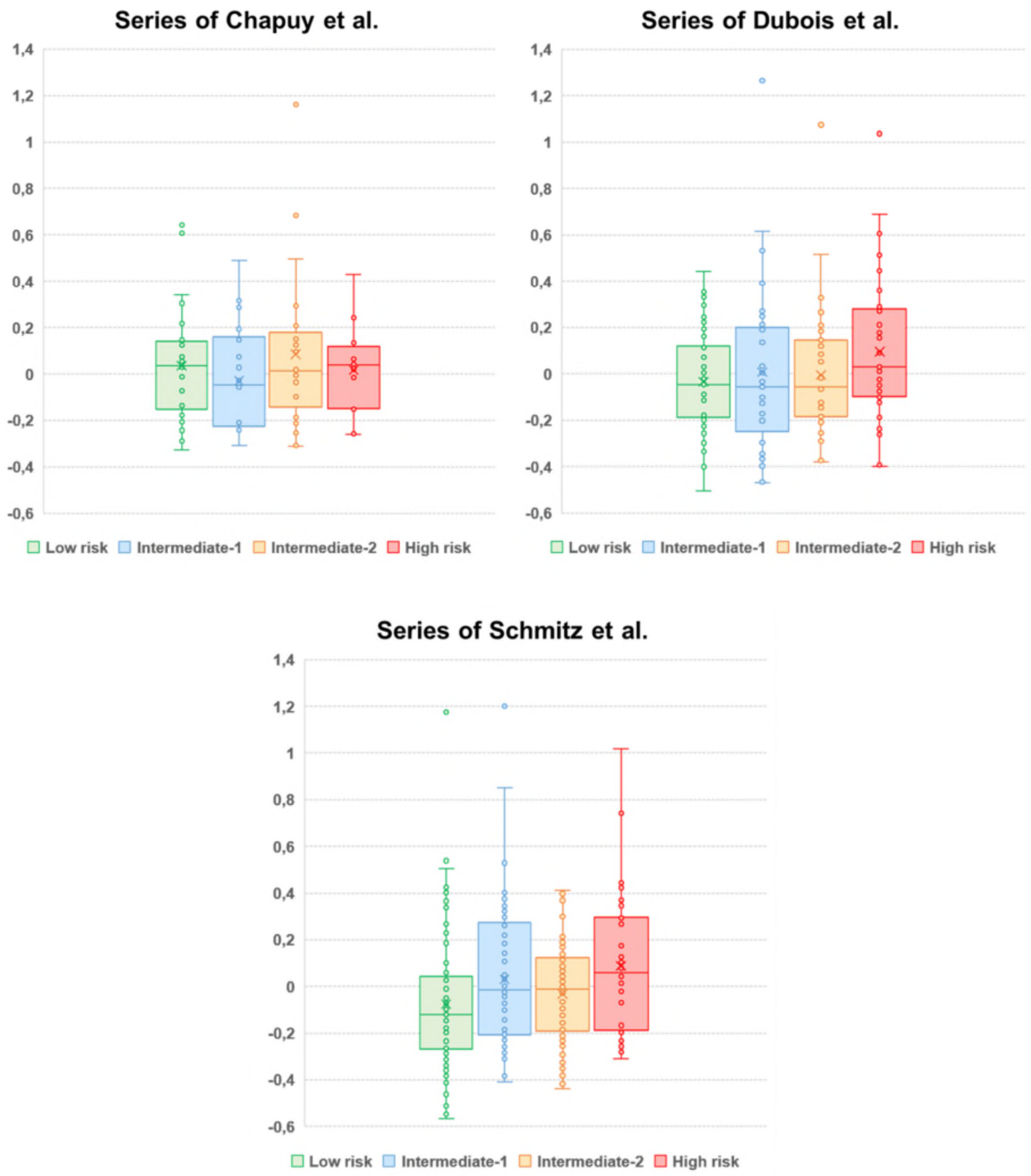


**Supplementary Fig. 30. Distribution of LCS scores among prognostic groups according to international prognostic index in three large DLBCL series.** Series from Chapuy et al: n=29 independent low-risk DLBCLs, n=22 independent intermediate-1 DLBCLs, n=21 independent intermediate-2 DLBCLs, and n=16 independent high-risk DLBCLs. Series from Dubois et al: n=51 independent low-risk DLBCLs, n=29 independent intermediate-1 DLBCLs, n=43 independent intermediate-2 DLBCLs, and n=45 high-risk DLBCLs. Series from Schmitz et al: n=85 independent low-risk DLBCLs, n=72 independent intermediate-1 DLBCLs, n=66 independent intermediate-2 DLBCLs, and n=43 independent high-risk DLBCLs. Box plots: the center line, box limits, whiskers and points represent the median, 25$^{th}$ and 75$^{th}$ percentile, 1.5x interquartile range and individual samples, respectively. Two-sided Student t-test. Source data are provided as a Source Data file.

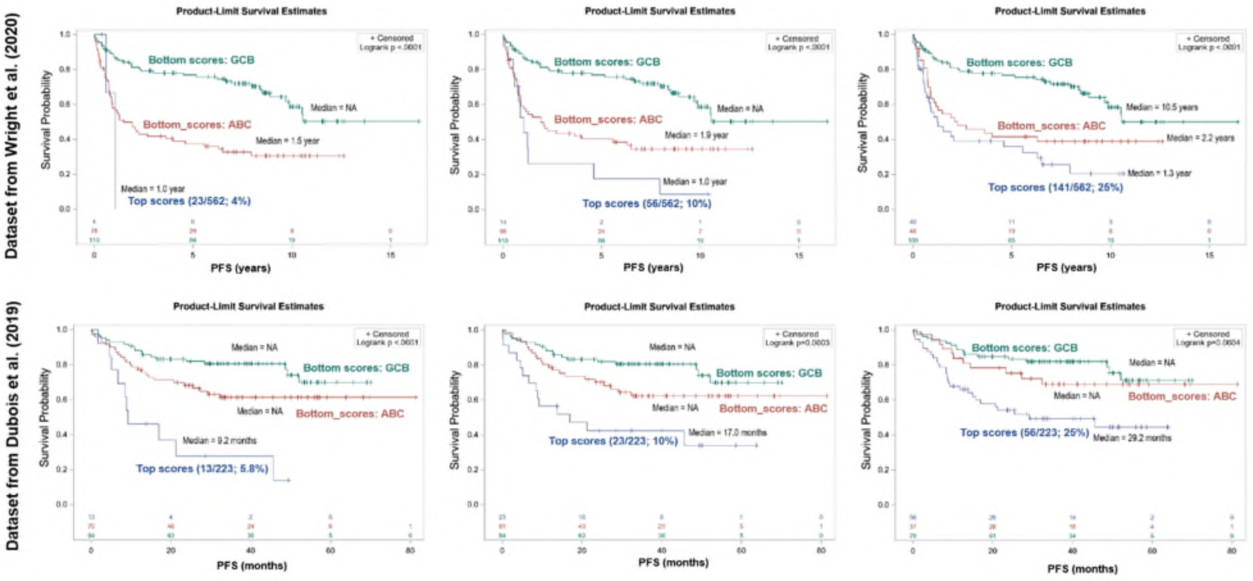


**Supplementary Fig. 31. Kaplan-Meier estimates of PFS for 785 patients from two combined DLBCL datasets with clinical annotations.** Comparative PFS between groups of patients determined according to progressive thresholds with top-LCS RS samples and the rest of the cohorts, according to COO. Upper panel: dataset from Wright and colleagues (PMID: 32289277): n=562 patients; p=1.2e-6 (top 25 LCS); p=1.9e-7 (top 10% LCS); p=8.5e-7 (top 25% LCS). Lower panel: dataset from Dubois and colleagues (PMID: 31648986): n=223 patients; p=1.6e-5 (top 25 LCS); p=3e-4 (top 10% LCS); p=4e-4 (top 25% LCS). Statistical comparisons were performed with Log-rank test. Bonferroni method was used for multitesting adjustments. COO: cell-of-origin; DLBCL: *de novo* diffuse large B-cell lymphoma. ABC: activated B-cell; COO: cell of origin; GCB: germinal center B-cell; LCS: linear classifier score; PFS: progression-free survival; RS: Richter syndrome.

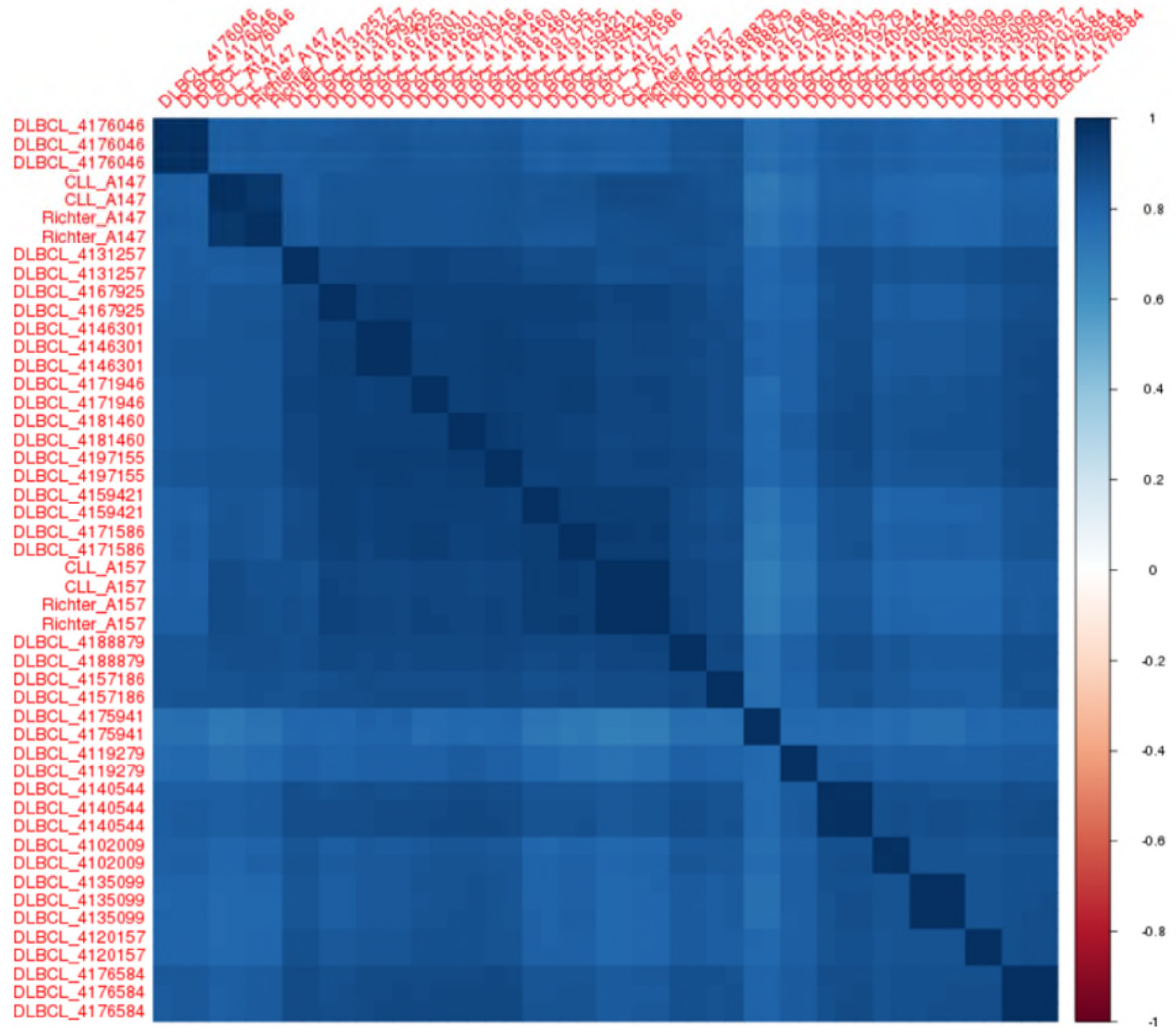


**Supplementary Fig. 32. Correlation heat map of all replicate samples in the FULL dataset after complete QC, SWAN normalization and extended checks.** SWAN: subset-quantile within array normalization; QC: quality control.

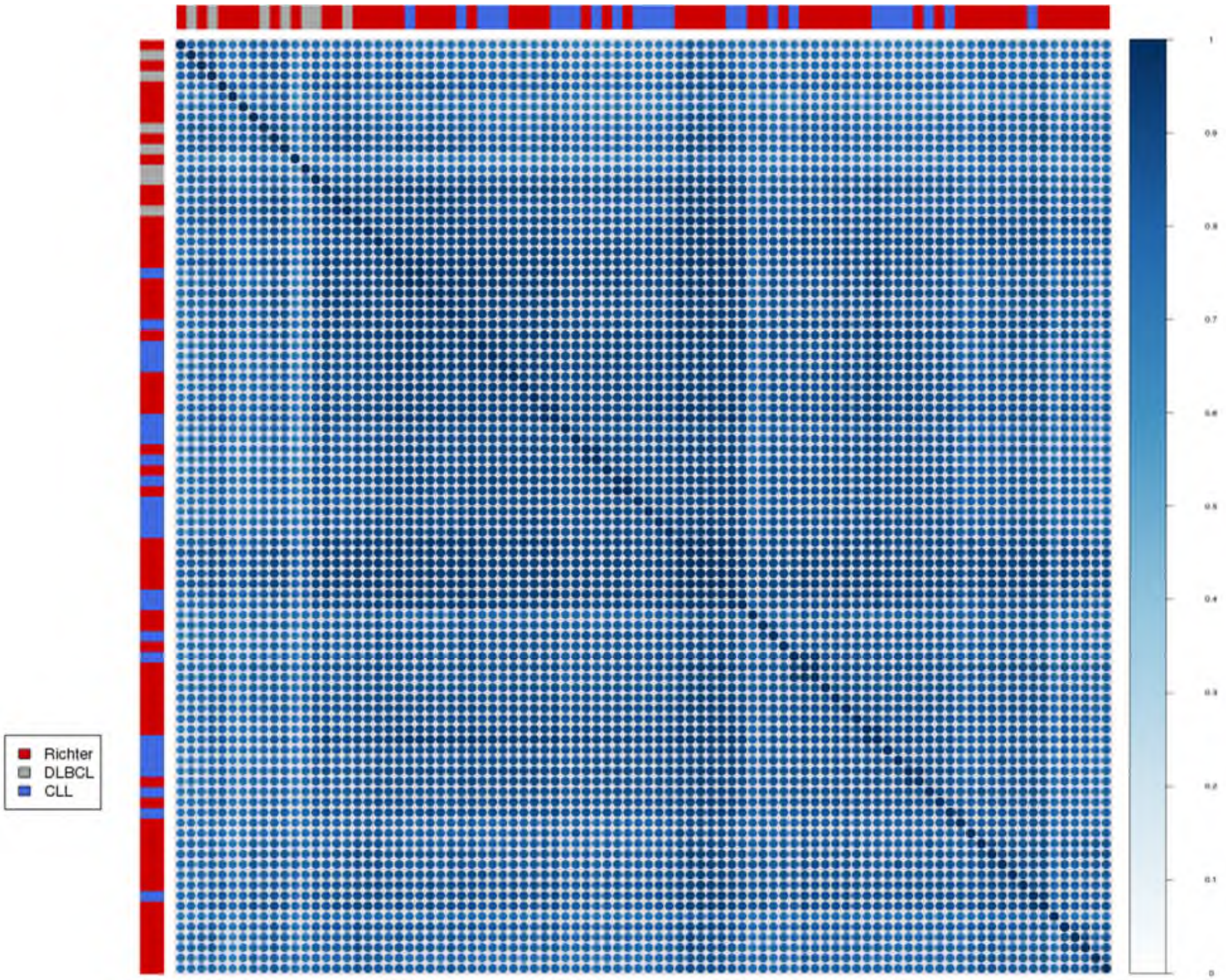


**Supplementary Fig. 33. Correlation heat map for the entire 90-sample EPIC dataset after SWAN normalization, extended QC and checks.** Pearson's correlation values: min = 0.64; median = 0.86; mean = 0.85. CLL: chronic lymphocytic leukemia; DLBCL: *de novo* diffuse large B-cell lymphoma; QC: quality control; RS: Richter syndrome; SWAN: Subset-quantile within array normalization.

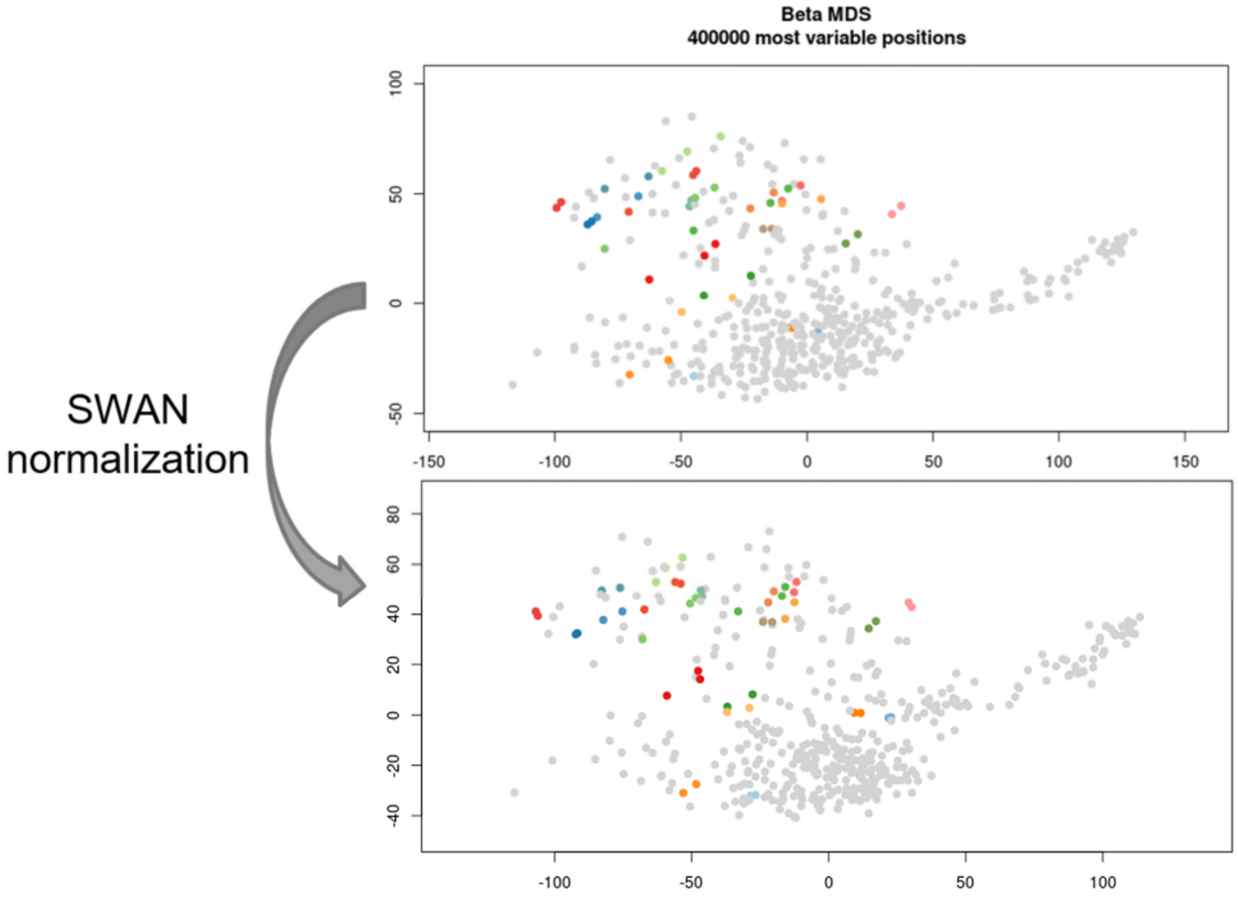


**Supplementary Fig. 34. Multi-dimensional scaling before and after normalization of the FULL dataset.** Color emphasis on the 7 *de novo* DLBCL replicates between French and German facilities, and 2 RS and 2 CLL replicates between French batches. Upper panel: before normalization, showing a small shift between many replicates, always in the same direction. Lower panel: after normalization, showing the correction of the shift, as seen here between replicate samples.

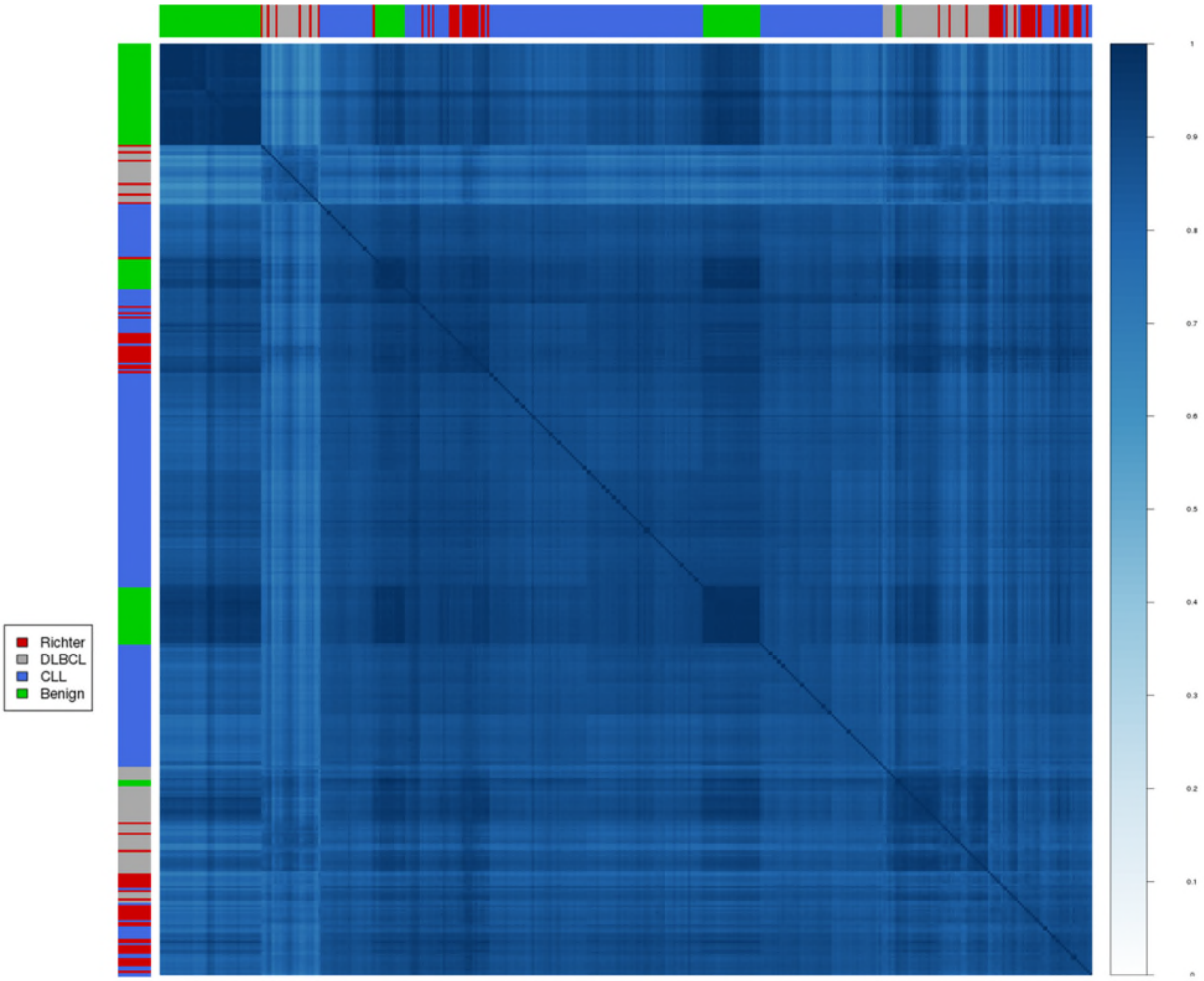


**Supplementary Fig. 35. Correlation heat map for the entire 433-sample FULL dataset after SWAN normalization, extended QC and checks.** Pearson's correlation values: min = 0.58; median = 0.88; mean = 0.87. Benign: normal B-cells spanning different stages of the B-lymphopoiesis; CLL: chronic lymphocytic leukemia; DLBCL: *de novo* diffuse large B-cell lymphoma; QC: quality control; RS: Richter syndrome; SWAN: subset-quantile within array normalization.

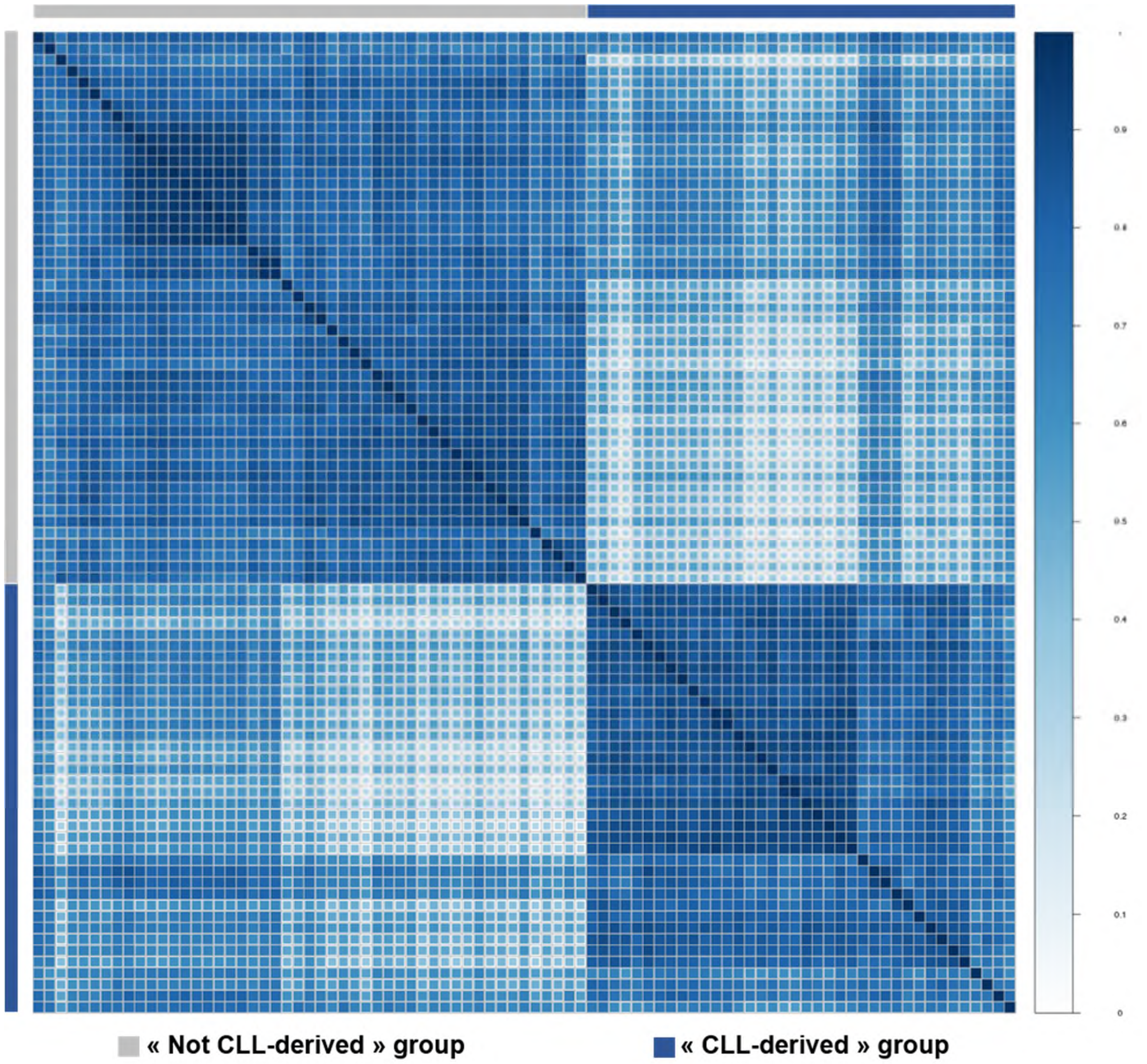


**Supplementary Fig. 36. Correlation matrix of the ranked 215-gene signature.** There is a clear discrimination between two groups according to this gene signature: i) the « CLL-derived group » is the CLL-derived RS signature-sharing samples, composed of CLLs + $_{low}$CLL-derived RS + $_{high}$CLL-derived RS, and ii) the « not CLL-derived group », composed of all other samples, naming de novo DLBCLs, tMZL (1 sample), tWM (1 sample), DLBCL-like RS and benigns. Benign: normal lymph node; CLL: chronic lymphocytic leukemia; DLBCL: diffuse large B-cell lymphoma; tMZL: transformed marginal zone lymphoma; tWM: transformed Waldenström Macroglobulinemia; RS: Richter syndrome.

# Supplemental methods

## *DNA and RNA extraction and qualification*

Nucleic acids were extracted from fresh frozen biopsies with separated Macherey Nagel extraction kits for DNA and RNA, respectively (Macherey-Nagel, Düren, Germany; cat. No. 740952.50 and 740955.50). DNA was available for 58 RS samples. DNA quality assessment was performed with TapeStation (Agilent, Santa-Clara, CA, USA), with genomics DNA screen tape (Agilent technologies; cat. No. 5067-5365 and 5067-5366) and RNA screen tape (Agilent technologies; cat. No. 5067-5577 and 5067-5578). RNA samples with a RNA integrity number > 8 were selected for transcriptome-Seq, all sequenced within a single batch. This protocol ensured the elimination of most sources of technical variations known to significantly affect transcriptomic measurements.

## IGHV *mutational status assessment*

IGHV mutational status was centrally assessed, according to ERIC (European Research Initiative on CLL) recommendations.[(1)] LymphoTrack IGH FR1/2/3 assay (Invivoscribe, San Diego, CA, USA; cat. no. 9-121-0129) and subsequent sequencing with MiSeq (Illumina) were used when no predominant VDJ rearrangement could be identified with GeneScan.

## *Targeted Next-Generation Sequencing*

Targeted NGS was performed using Ampli-Seq (Illumina; cat. no. 20020495 and cat. no. 20019102) method according to the manufacturer’s protocol. A 13-gene panel was used, including *TP53* (exons 2-10), *SF3B1* (exons 12-16), *NOTCH1* (exon 34), *ATM* (exons 2-

63), *BIRC3* (exons 6 and 7), *MYD88* (exons 3 and 5), *FBXW7* (exons 1-11), *POT1* (exons 4-7 and 9), *XPO1* (exons 15 and 16), *RPS15* (exons 3 and 4), *NFKBIE* (exons 1-6), *EGR2* (exons 1 to 3), *BRAF* (exon 15). Sequences were aligned, and mutation calling was performed using a custom bioinformatics pipeline.[2] A threshold of 5% variant allelic fraction was chosen to retain mutations.

## *Illumina methylation EPIC 850K microarray*

The EPIC microarray interrogates 850K CpG sites selected among those described in cancer databases, allowing a large-scale methylome study including proximal promoter regions, distal regulatory regions, and gene bodies.[3, 4] Six hundred nanograms of genomic DNA were converted by bisulfite using the *EZ DNA Methylation kit* (Zymo Research, Proteigene, Saint-Marcel, France; cat. no. D5001). DNA conversion by bisulfite was tested by specific methylation PCR (MSP) with primers targeting the CpG island (unmethylated) of the paternal allele of *SNRPN* gene using ZymoTaq Premix (Zymo Research; cat. no. E2003). Bisulfite-converted DNA was hybridized to *Infinium Methylation EPIC array* (Illumina, San Diego, CA, USA; cat. no. WG-317-1002 and cat. no. 20020531), referred to as the EPIC 850K platform, following the manufacturer’s instructions, allowing to determine the DNA methylation status of 866,562 CpG sites. Probe annotation information, including sequence and chromosome location for the EPIC 850K array was retrieved from the MethylationEPIC_v-1-0 manifest file.

## Copy number variations and detection of CpG deletions.

Copy number variation (CNV) analyses, gene and promoter regions probe retrieval, β-value visualization and CpG deletion detections were performed on the EPIC dataset with

the R package SeSAMe.[5] The *openSesame* pipeline was used with default parameters for background subtraction with *noobsb*, dye bias correction with *dyeBiasCorr*, probe masking on quality and detection. β-values were finally obtained by using *getBetas* with sum.TypeI = TRUE as a parameter to rescue probes with SNP hitting the extension base and hence switching color channel.[6] Promoter regions were plotted using β-values with *visualizeGene* and *visualizeRegion*, with deleted CpGs marked as masked or removed. CNVs were normalized against a copy-number-normal data set (EPIC.5. normal; available in the package), computed with the *cnSegmentation* function, and visualized with *visualizeSegments*. DNAcopy segmentation files were generated for all Richter samples, with image files created for each chromosome.

## *Transcriptomics*

**Library preparation and RNA-sequencing.** According to the manufacturer's instructions, total RNA-Seq libraries were generated from 500 ng of total RNA using *TruSeq Stranded Total RNA LT Sample Prep Kit with Ribo-Zero Gold* (Illumina; cat. no. RS122-2301 and RS-122-2302). Final cDNA libraries were checked for quality and quantified using capillary electrophoresis. Sequencing was then performed with Hiseq 4000 sequencing using a 1x50 bases protocol.

**Variant calling from expressed transcripts.** SNPs and small indels for each sample were called against reference hg38 from pileups generated with samtools with parameters -A -C 50 -q 10 -Q 30 -m 2 to discard bad nucleotide or sequence alignment quality. Multiallelic variants were called with bcftools v1.3.1 [http://github.com/samtools/bcftools] with mutation rate set at 1/1000 and default ploidy for GRCh38 and were then filtered to

exclude low depth of coverage (DP < 10) and quality biased (QUAL < 10) calls. Samples were merged, indels normalized to the reference sequence, and multiallelic sites split into multiple biallelic entries, then genotypes and call statistics were re-evaluated for every locus. Low-quality positions (based on reported statistical tests) were finally discarded and replaced with missing genotypes. Variants were annotated with Annovar version 2019-10-24,[7] with databases among avsnp147, cosmic70, clinvar_20160302, dbscsnv11 and dbnsfp30a, Ensembl and refSeq annotations.

## ICGC MMML-seq consortium members who participated to this work:

Ole Ammerpohl[(1)]

Stephan Bernhart[(2)]

Markus Kreuz[(3)]

Peter Lichter[(4)]

German Ott[(5)]

Andreas Rosenwald[(6)]

Reiner Siebert[(1)]

Stephan Stilgenbauer[(7)]

(1)Institute of Human Genetics, Ulm University & Ulm University Medical Center, Ulm, Germany

(2)Bioinformatics Group, Department of Computer Science and Interdisciplinary Center for Bioinformatics, Leipzig University, Germany.

(3) Fraunhofer Institute for Cell Therapy and Immunology IZI, Leipzig, Germany.

(4)Division of Molecular Genetics, German Cancer Research Center (DKFZ), Heidelberg, Germany.

(5)Department of Clinical Pathology, Robert-Bosch Krankenhaus, and Dr. Margarete Fischer-Bosch Institute for Clinical Pharmacology, Stuttgart, Germany.

(6)Institute of Pathology, University Hospital of Würzburg, Germany.

(7)Division of CLL. Department of Internal Medicine III, Ulm University, Ulm, Germany.

# ICGC MMML-seq consortium members:

### Full list of members of the ICGC MMML-Seq consortium

*Coordination (C1):* Reiner Siebert[1,2], Susanne Wagner[2], Andrea Haake[2], Julia Richter[2,3], Gesine Richter[2]
*Data Center (C2):* Roland Eils[4,5], Chris Lawerenz[4], Jürgen Eils[4], Jules Kerssemakers[4], Christina Jaeger-Schmidt[4], Ingrid Scholz[4]
*Clinical Centers (WP1):* Anke K. Bergmann[2, 6], Christoph Borst[7], Friederike Braulke[8], Birgit Burkhardt[9,10], Alexander Claviez[6], Martin Dreyling[11], Sonja Eberth[11], Hermann Einsele[12], Norbert Frickhofen[13], Siegfried Haas[7], Martin-Leo Hansmann[14], Dennis Karsch[15], Nicole Klepl[8], Michael Kneba[15], Jasmin Lisfeld[9], Luisa Mantovani-Löffler[16], Marius Rohde[9], German Ott[17], Christina Stadler[8], Peter Staib[18], Stephan Stilgenbauer[19], Lorenz Trümper[8] , Thorsten Zenz[20]
*Normal Cells (WPN):* Martin-Leo Hansmann[14], Dieter Kube[8], Ralf Küppers[21], Marc Weniger[21]
*Pathology and Analyte Preparation (WP2-3):* Siegfried Haas[7], Michael Hummel[22], Wolfram Klapper[3], Ulrike Kostezka[23], Dido Lenze[22], Peter Möller[24], Andreas Rosenwald[25], German Ott[17], Monika Szczepanowski[3]
*Sequencing and genomics (WP4-7):* Ole Ammerpohl[1,2], Sietse M. Aukema[2,3], Vera Binder[26], Arndt Borkhardt[26], Andrea Haake[2], Jessica I. Hoell[26]; Ellen Leich[25], Peter Lichter[27], Cristina López[1,2], Inga Nagel[2], Jordan Pischimariov[25], Bernhard Radlwimmer[27], Julia Richter[2,3], Philip Rosenstiel[28], Andreas Rosenwald[25], Markus Schilhabel[28], Stefan Schreiber[29], Inga Vater[2], Rabea Wagener[1,2], Reiner Siebert[1,2]
*Bioinformatics (WP8-9):* Stephan H. Bernhart[30-32,] Hans Binder[30,31], Benedikt Brors[33], Gero Doose[30-32], Roland Eils[4,5], Steve Hoffmann[30-32], Lydia Hopp[30], Daniel Hübschmann[4,5,34], Kortine Kleinheinz[4,5], Helene Kretzmer[30-32], Markus Kreuz[35], Jan Korbel[36], David Langenberger[30-32], Markus Loeffler[35], Maciej Rosolowski[35], Matthias Schlesner[4,37] , Peter F. Stadler[30-32,38-40], Stephanie Sungalee[36]

[1]Institute of Human Genetics, University of Ulm and University Hospital of Ulm, Ulm, Germany
[2]Institute of Human Genetics, Christian-Albrechts-University, Kiel, Germany;
[3]Hematopathology Section, Institute of Pathology, Christian-Albrechts-University, Kiel, Germany;
[4]Division of Theoretical Bioinformatics (B080), German Cancer Research Center (DKFZ), Heidelberg, Germany;
[5]Department for Bioinformatics and Functional Genomics, Institute of Pharmacy and Molecular Biotechnology and Bioquant, University of Heidelberg, Heidelberg, Germany;
[6]Department of Pediatrics, University Hospital Schleswig-Holstein, Campus Kiel, Kiel, Germany;
[7]Department of Internal Medicine/Hematology, Friedrich-Ebert-Hospital, Neumünster;
[8]Department of Hematology and Oncology, Georg-August-University of Göttingen, Göttingen, Germany;
[9]University Hospital Muenster - Pediatric Hematology, and Oncology, Muenster Germany;
[10]University Hospital Giessen, Pediatric Hematology, and Oncology, Giessen, Germany;
[11]Department of Medicine III - Campus Grosshadern, University Hospital Munich, Munich, Germany;
[12]University Hospital Würzburg, Department of Medicine and Poliklinik II, University of Würzburg, Würzburg;
[13]Department of Medicine III, Hematology and Oncology, Dr. Horst-Schmidt-Kliniken of Wiesbaden, Wiesbaden;
[14]Senckenberg Institute of Pathology, University of Frankfurt Medical School, Frankfurt am Main, Germany
[15]Department of Internal Medicine II: Hematology and Oncology, University Medical Centre, Campus Kiel, Kiel;
[16]Hospital of Internal Medicine II, Hematology and Oncology, St-Georg Hospital Leipzig, Leipzig, Germany;
[17]Department of Clinical Pathology, Robert-Bosch-Hospital, Stuttgart, Germany;
[18]Clinic for Hematology and Oncology, St.-Antonius-Hospital, Eschweiler;
[19]Department for Internal Medicine III, University of Ulm and University Hospital of Ulm, Ulm, Germany
[20]National Centre for Tumor Disease, Heidelberg, Germany;
[21]Institute of Cell Biology (Cancer Research), University of Duisburg-Essen, Duisburg-Essen, Medical School, Essen, Germany;
[22]Institute of Pathology, Charité – University Medicine Berlin, Berlin, Germany;
[23]Comprehensive Cancer Center Ulm (CCCU), University Hospital Ulm, Ulm, Germany;
[24]Institute of Pathology, University of Ulm and University Hospital of Ulm, Ulm;

[25]Institute of Pathology, Comprehensive Cancer Center Mainfranken, University of Wurzburg, Germany;
[26]Department of Pediatric Oncology, Hematology and Clinical Immunology, Heinrich-Heine-University, Düsseldorf, Germany;
[27]German Cancer Research Center (DKFZ), Division of Molecular Genetics, Heidelberg, 69120, Germany;
[28]Institute of Clinical Molecular Biology, Christian-Albrechts-University, Kiel, Germany;
[29]Department of General Internal Medicine, University Kiel, Kiel, Germany;
[30]Interdisciplinary Center for Bioinformatics, University of Leipzig, Leipzig, Germany;
[31]Bioinformatics Group, Department of Computer, University of Leipzig, Leipzig, Germany; [32]Transcriptome Bioinformatics, LIFE Research Center for Civilization Diseases, University of Leipzig, Leipzig, Germany;
[33]Division of Applied Bioinformatics (G200), German Cancer Research Center (DKFZ), Heidelberg, Germany
[34]Department of Pediatric Immunology, Hematology and Oncology, University Hospital, Heidelberg, Germany
[35]Institute for Medical Informatics Statistics and Epidemiology, University of Leipzig, Leipzig, Germany;
[36]EMBL Heidelberg, Genome Biology, Heidelberg, Germany;
[37]Bioinformatics and Omics Data Analytics (B240), German Cancer Research Center (DKFZ), Heidelberg, Germany;
[38]RNomics Group, Fraunhofer Institute for Cell Therapy and Immunology IZI, Leipzig, Germany
[39]Santa Fe Institute, Santa Fe, New Mexico, United States of America
[40]Max-Planck-Institute for Mathematics in Sciences, Leipzig, Germany.

## MMML-seq members:

*Pathology group and analytes preparation:* Thomas F.E. Barth[1], Heinz-Wolfram Bernd[2], Sergio B. Cogliatti[3], Alfred C. Feller[2], Martin L. Hansmann[4], Michael Hummel[5], Wolfram Klapper[6], Dido Lenze[5], Peter Möller[1], Hans-Konrad Müller-Hermelink[7], German Ott[7], Andreas Rosenwald[7], Harald Stein[5], Monika Szczepanowski[6], Hans-Heinrich Wacker[6].

*Genetics group:* Thomas F.E. Barth[1], Petra Behrmann[8], Peter Daniel[10], Judith Dierlammm[8], Eugenia Haralambieva[7], Lana Harder[11], Paul-Martin Holterhus[12], Ralf Küppers[13], Dieter Kube[13], Peter Lichter[14], Jose I. Martín-Subero[11], Peter Möller[1], Eva M. Murga-Peñas[9], German Ott[7], Christiane Pott[16], Armin Pscherer[15], Andreas Rosenwald[7], Carsten Schwaenen[17], Reiner Siebert[18], Heiko Trautmann[16], Martina Vockerodt[19], Swen Wessendorf[16].

*Bioinformatics group:* Stefan Bentink[20], Hilmar Berger[21], Dirk Hasenclever[21], Markus Kreuz[21], Markus Loeffler[21], Maciej Rosolowski[21], Rainer Spang[20].

*Project coordination:* Benjamin Stürzenhofecker[14], Lorenz Trümper[14], Maren Wehner[14].

*Steering committee:* Markus Loeffler[21], Reiner Siebert[18], Harald Stein[5], Lorenz Trümper[14].

[1]Institute of Pathology, University Hospital of Ulm, Ulm, Germany;
[2]Institute of Pathology, University Hospital Schleswig-Holstein Campus Lübeck, Lübeck, Germany;
[3]Institute of Pathology, Kantonsspital St. Gallen, St.Gallen, Switzerland;
[4]Institute of Pathology, University Hospital of Frankfurt, Frankfurt, Germany;
[5]Institute of Pathology, Campus Benjamin Franklin, Charité–Universitätsmedizin Berlin, Berlin, Germany;
[6]Institute of Hematopathology, University Hospital Schleswig-Holstein Campus Kiel/ Christian-Albrechts University Kiel, Kiel, Germany;
[7]Institute of Pathology, University of Würzburg, Würzburg, Germany;
[8]Cytogenetic and Molecular Diagnostics, Internal Medicine III, University Hospital of Ulm, Ulm, Germany;
[9]University Medical Center Hamburg-Eppendorf, Hamburg, Germany;
[10]Department of Hematology, Oncology and Tumor Immunology, University Medical Center Charité, Berlin, Germany;
[11]Institute of Human Genetics, University Hospital Schleswig-Holstein Campus Kiel/Christian-Albrechts University Kiel, Kiel, Germany;
[12]Division of Pediatric Endocrinology and Diabetes, Department of Pediatrics, University Hospital Schleswig- Holstein Campus Kiel / Christian-Albrechts University Kiel, Kiel, Germany;
[13]Institute for Cell Biology (Tumor Research), University of Duisburg-Essen, Essen, Germany;
[14]Department of Hematology and Oncology, Georg-August University of Göttingen, Göttingen, Germany;
[15]German Cancer Research Center (DKFZ), Heidelberg, Germany;
[16]Second Medical Department, University Hospital Schleswig-Holstein Campus Kiel/ Christian-Albrechts University Kiel, Kiel, Germany;
[17]Cytogenetic and Molecular Diagnostics, Internal Medicine III, University Hospital of Ulm, Ulm, Germany;
[18]Institute of Human Genetics, University of Ulm and University Hospital of Ulm, Ulm, Germany
[19]Department of Pediatrics I, Georg-August University of Göttingen, Göttingen, Germany;
[20]Institute of Functional Genomics, University of Regensburg, Regensburg, Germany;
[21]Institute for Medical Informatics, Statistics and Epidemiology, University of Leipzig, Leipzig, Germany.

# References

1. Ghia, P. et al. ERIC recommendations on IGHV gene mutational status analysis in chronic lymphocytic leukemia. Leukemia **21**,1-3 (2007).

2. Tausch, E. et al. Prognostic and predictive impact of genetic markers in patients with CLL treated with obinutuzumab and venetoclax. Blood **135**, 2402-2412 (2020).

3. Bibikova, M. et al. High density DNA methylation array with single CpG site resolution. Genomics **98**, 288-295 (2011).

4. Pidsley, R. et al. Critical evaluation of the Illumina MethylationEPIC BeadChip microarray for whole-genome DNA methylation profiling. Genome Biol. **17**, 208 (2016).

5. Zhou, W., Triche, T.J., Laird, P.W.& Shen, H. SeSAMe: reducing artifactual detection of DNA methylation by Infinium BeadChips in genomic deletions. Nucleic Acids Res. **46**, :e123 (2018).

6. Zhou, W., Laird, P.W. & Shen, H. Comprehensive characterization, annotation and innovative use of Infinium DNA methylation BeadChip probes. Nucleic Acids Res. **45**, e22 (2017).

7. Wang, K., Li, M. & Hakonarson, H. ANNOVAR: functional annotation of genetic variants from high-throughput sequencing data. Nucleic Acids Res. **38**, e164 (2010).
