# Supplementary material for: Molecular characterization of Richter syndrome identifies de novo diffuse large B-cell lymphomas with poor prognosis
Source: Nat Commun. 2023 Jan 19;14:309. doi: 10.1038/s41467-022-34642-6 (PMC9852595; doi:10.1038/s41467-022-34642-6)
Supplement: Supplementary file 3 — Description to Additional Supplementary Information [file 41467_2022_34642_MOESM3_ESM.pdf]

Legends for supplementary dataset

Main title: Supplementary Data

Supplementary data 1. Functional annotations of genes regulated by regulatory sequences located in Differentially Methylated Regions (DMRs) between RS and de novo DLBCL.

Supplementary data 2. Functional annotations of genes regulated by regulatory sequences located in Differentially Methylated Regions (DMRs) between RS and CLL.

Supplementary data 3. Functional annotations of the transcriptome K-means cluster C1, with a down-regulated CLL-derived Richter Syndrome signature, along with term enrichments over several gene ontology, pathway, regulatory targets, disease and crowd databases.

Supplementary data 4. Functional annotations of the transcriptome K-means cluster C6, with an upregulated CLL-derived Richter Syndrome signature, along with term enrichments over several gene ontology, pathway, disease and crowd databases.

Supplementary data 5. Functional analyses of an extracted list of 666 genes which expression level was negatively correlated with methylation level of regulatory sequences ($p < 0.01$; Spearman's rho < -1/3; at least 3 hits in the same regulatory region).

Supplementary data 6. Functional analyses of an extracted list of 234 genes which expression level was positively correlated with methylation level of regulatory sequences ($p < 0.01$; Spearman's rho < -1/3; at least 3 hits in the same regulatory region).

Supplementary data 7. Overrepresented ontologies, pathways and regulatory targets obtained from the functional network linking 861 significant genes extracted with the methylome/transcriptome integrative approach.

Supplementary data 8. Functional annotation of the 156 transcription factors with overrepresented families, functions and encoded protein domains.

Supplementary data 9. Relative expression level for the 215 genes in the CLL-derived RS signature versus DLBCLs-like RS.

Supplementary data 10. Genotype check for RS samples with methylation data. Genotype match: yes or unknown (either methylome or transcriptome positions are not usable) or not applicable (either methylome or transcription data are unavailable).

Supplementary data 11. Technical and biological covariate table for all 433 samples from the FULL methylation dataset.
