# Supplementary material for: Molecular characterization of Richter syndrome identifies de novo diffuse large B-cell lymphomas with poor prognosis
Source: Nat Commun. 2023 Jan 19;14:309. doi: 10.1038/s41467-022-34642-6 (PMC9852595; doi:10.1038/s41467-022-34642-6)
Supplement: Supplementary file 5 — Reporting Summary [file 41467_2022_34642_MOESM5_ESM.pdf]

nature portfolio

Corresponding author(s): Pr Julien BROSÉUS
Pr Stephan STILGENBAUER

Last updated by author(s): Oct 25, 2022

Reporting Summary

Nature Portfolio wishes to improve the reproducibility of the work that we publish. This form provides structure for consistency and transparency in reporting. For further information on Nature Portfolio policies, see our Editorial Policies and the Editorial Policy Checklist.

Statistics

For all statistical analyses, confirm that the following items are present in the figure legend, table legend, main text, or Methods section.

n/a	Confirmed	
☐	☒	The exact sample size (*n*) for each experimental group/condition, given as a discrete number and unit of measurement
☐	☒	A statement on whether measurements were taken from distinct samples or whether the same sample was measured repeatedly
☐	☒	The statistical test(s) used AND whether they are one- or two-sided<br>*Only common tests should be described solely by name; describe more complex techniques in the Methods section.*
☐	☒	A description of all covariates tested
☐	☒	A description of any assumptions or corrections, such as tests of normality and adjustment for multiple comparisons
☐	☒	A full description of the statistical parameters including central tendency (e.g. means) or other basic estimates (e.g. regression coefficient) AND variation (e.g. standard deviation) or associated estimates of uncertainty (e.g. confidence intervals)
☐	☒	For null hypothesis testing, the test statistic (e.g. *F*, *t*, *r*) with confidence intervals, effect sizes, degrees of freedom and *P* value noted<br>*Give P values as exact values whenever suitable.*
☒	☐	For Bayesian analysis, information on the choice of priors and Markov chain Monte Carlo settings
☒	☐	For hierarchical and complex designs, identification of the appropriate level for tests and full reporting of outcomes
☐	☒	Estimates of effect sizes (e.g. Cohen's *d*, Pearson's *r*), indicating how they were calculated

Our web collection on statistics for biologists contains articles on many of the points above.

Software and code

Policy information about availability of computer code

Data collection	Expression matrices and metadata from GEO were downloaded with the R package GEOquery (v2.62.2; Davis S, Meltzer PS. Bioinformatics. 2007;23(14):1846-7).<br>The following databases were used: Ensembl (https://www.ensembl.org/index.html; release 107; Cunningham F, et al. Nucleic Acids Res. 2019;47(D1):D745-D51); UCSC; Gene Expression Omnibus curated database (https://www.ncbi.nlm.nih.gov/geo/)<br>GRCh38 annotations (version 90); refSeq (https://www.ncbi.nlm.nih.gov/refseq/); Open Targets platform (https://platform.opentargets.org/; Ochoa D, et al. Nucleic Acids Res. 2021;49(D1):D1302-D10); ENCODE (v128.1; https://www.encodeproject.org/); JASPAR (9th release; https://jaspar.genereg.net/).<br><br>The protocols are fully detailed in the Online Methods section of the manuscript
Data analysis	Analyzes were run under R 3.6 with Bioconductor 3.10 and the corresponding versions of the following R packages : biomaRt (Durinck S, et al. Nat Protoc. 2009;4(8):1184-91), SWAN (Maksimovic J, et al. Genome Biol. 2012;13(6):R44), ReactomePA (Yu G, et al. Mol Biosyst. 2016;12(2):477-9), DMRcate, imputePCA, minfi (Aryee MJ, et al. Bioinformatics. 2014;30(10):1363-9), CMplot, missMDA (Josse J, François H. Journal of Statistical Software; 2016. p. 1-31), corrplot, OmicsPrint (van Iterson M, et al. Bioinformatics. 2018;34(12):2142-3), SeSAMe (Zhou W, et al. Nucleic Acids Res. 2018;46(20):e123), missMethyl (Phipson B, et al. Bioinformatics. 2016;32(2):286-8), liftOver, XRG, factoMineR, factoextra, NMF, ComplexHeatMap, TMM (Robison et al. Genome Biology. 2010).<br><br>The following open source programs have been used: STRING (Szklarczyk D, et al. Nucleic Acids Res. 2019;47(D1):D607-D13; https://string-db.org/); NetworkAnalyst (Zhou G, et al. Nucleic Acids Res. 2019;47(W1):W234-W41; www.networkanalyst.ca); Enrichr (Kuleshov MV, et al. Nucleic Acids Res. 2016;44(W1):W90-7; https://maayanlab.cloud/Enrichr) , FastQC v0.11.5 (http://www.bioinformatics.babraham.ac.uk/projects/fastqc/), HISAT2 (v2.0.4; Kim D, et al. Nat Methods. 2015;12(4):357-60.); Samtools (v1.3.1; http://github.com/samtools/samtools),

Picard (v1.13; https://broadinstitute.github.io/picard/), StringTie (v2.1.0; Pertea M, et al. Nat Biotechnol. 2015;33(3):290-5), bcftools (v1.3.1; http://github.com/samtools/bcftools), Annovar (version 2019-10-24; Wang K, et al. Nucleic Acids Res. 2010;38(16):e164).

Custom code developed for this study is available on the GitHub platform, at the following link: https://github.com/zetcheuv/RichterOmicsCode

For manuscripts utilizing custom algorithms or software that are central to the research but not yet described in published literature, software must be made available to editors and reviewers. We strongly encourage code deposition in a community repository (e.g. GitHub). See the Nature Portfolio guidelines for submitting code & software for further information.

Data

Policy information about availability of data

All manuscripts must include a data availability statement. This statement should provide the following information, where applicable:

- Accession codes, unique identifiers, or web links for publicly available datasets
- A description of any restrictions on data availability
- For clinical datasets or third party data, please ensure that the statement adheres to our policy

Methylation data from Richter samples are accessible upon request via the European Genome-Phenome Archive (Study EGAS00001005495; accession number EGAD00010002194; https://ega-archive.org/datasets/EGAD00010002194). Transcriptomic data are accessible upon request via the European Genome-Phenome Archive (Study EGAS00001005495; accession number EGAD00001007922; https://ega-archive.org/datasets/EGAD00001007922). Targeted NGS data are accessible upon request via the European Genome-Phenome Archive (Study EGAS00001005495; accession number EGAD00001009509). The raw data are protected and available under restricted access. Clinical and genomic data can be obtained by contacting the data access committee, according to the European Genome-Phenome Archive's procedure. Data access will be granted if their use complies with the data use conditions, including a commitment to strictly use these data for clearly identified academic research programs and according to good practice recommendations. The Data Access Committee will respond to requests within 2 weeks. Once access to the data is granted, these are available until the end of the research program they support. Source data are provided with this paper.

Previously published DNA methylation datasets from the ICGC MMML-seq consortium that were used in this study are available upon request from the data access committee at the ICGC MMML-seq consortium data portal (https://dcc.icgc.org/).

Following datasets were retrieved from GEO: GSE103265 (https://www.ncbi.nlm.nih.gov/geo/query/acc.cgi?acc=GSE103265), GSE10846 (https://www.ncbi.nlm.nih.gov/geo/query/acc.cgi?acc=GSE10846), GSE98588 (https://www.ncbi.nlm.nih.gov/geo/query/acc.cgi?acc=GSE98588), and GSE87371 (https://www.ncbi.nlm.nih.gov/geo/query/acc.cgi?acc=GSE87371).

Human research participants

Policy information about studies involving human research participants and Sex and Gender in Research.

Reporting on sex and gender

The terms "sex" and "gender" were carrefully used, according to recommendations and requirements of Nature journals.
No findings apply to one sex or gender.
Sex or gender were not considered in study design.
Sex and gender were determined based on self reporting and consent were obtained for sharing these data.
Overall numbers for patients diagnosed with Richter syndrome: males 39/58; females 19/58.

Population characteristics

The characteristics of the patients with chronic lymphocytic leukemia, de novo diffuse large B-cell lymphoma and Richter syndrome included in this study are similar to previous clinical descriptions. This cohort can be considered representative of these disease groups. Patients were recruited regardless of age, gender or other clinical and biological features, except stringent diagnosis criteria from the World Health Organization classification. The analyzed cohorts are representative of the studied diseases and cover the different treatment modalities.
Main clinical and biological variables were reported in a custom database that was extensively used throughout the study, paticularly for covariate correction in statistical models, and classification checks. Access and use of clinical data was allowed by dedicated academic legal entites.

Recruitment

Retrospective recruitment according to sample availability and high quality standards exigible for accurate omic studies.

Ethics oversight

This study complies with all relevant ethical regulations and we have obtained written informed consent for all participants. No compensation was provided. We obtained consent to use and publish information that identifies individuals, including indirect identifiers such as gender and age. Individuals recruited for this study can no longer be identified by the information provided, due to sample anonymization and processing of the genomic data. All procedures were in accordance with Helsinki declaration. Study protocol was approved by the Institutional Review Boards and Ethics Committees of Nancy, Kiel (#A150/10), Ulm (#349/11; #459/19 and #96/08) and Barcelona university hospitals, and by the French national ethics committee (Comité de Protection des Personnes Ouest IV 09/05/2017).

Note that full information on the approval of the study protocol must also be provided in the manuscript.

Field-specific reporting

Please select the one below that is the best fit for your research. If you are not sure, read the appropriate sections before making your selection.

☒ Life sciences ☐ Behavioural & social sciences ☐ Ecological, evolutionary & environmental sciences

Life sciences study design

All studies must disclose on these points even when the disclosure is negative.

Sample size	No statistical methods were used for sample size determination. We included all patients with suitable material available.<br>These sample sizes were considered sufficient, since they were comparable or higher to those reported in previous reference studies (Rinaldi et al. BJH 2013; Nadeu et al. Nat Med 2022). The analyzed cohort is representative of the studied diseases and cover the different treatment modalities.
Data exclusions	Data exclusion was performed for methylation and RNA-seq data, as detailed in the respective methods. Briefly, a few samples were discarded during an extensive quality control procedure.
Replication	DNA-methylation patterns and gene expression profiles obtained from the same samples with 2 orthogonal techniques were highly (> 95%) concordant. Statistical classifiers were devised to be robust and reproducible and were also successfully applied to large publicly available datasets. A validation cohort (n=52 samples) was built to perform a second EPIC 850K experiment and confirm DNA methylation-based classifier ability to discriminate between clonally related and clonally unrelated RS.
Randomization	Patients were allocated to the different groups according to the 2008 World Health Organization diagnostic criteria for chronic lymphocytic leukemia, de novo diffuse large B-cell lymphoma and Richter syndrome.<br>Covariates control was not relevant in this study since this is a retrospective biological study comparing DNA methylation and gene-expression data of Richter syndrome, de novo DLBCL and CLL.
Blinding	Not relevant for this study, since this is a retrospective biological study.

Reporting for specific materials, systems and methods

We require information from authors about some types of materials, experimental systems and methods used in many studies. Here, indicate whether each material, system or method listed is relevant to your study. If you are not sure if a list item applies to your research, read the appropriate section before selecting a response.

Materials & experimental systems

n/a	Involved in the study	
☒	☐	Antibodies
☒	☐	Eukaryotic cell lines
☒	☐	Palaeontology and archaeology
☒	☐	Animals and other organisms
☐	☒	Clinical data
☒	☐	Dual use research of concern

Methods

n/a	Involved in the study	
☒	☐	ChIP-seq
☒	☐	Flow cytometry
☒	☐	MRI-based neuroimaging

Clinical data

Policy information about clinical studies

All manuscripts should comply with the ICMJE guidelines for publication of clinical research and a completed CONSORT checklist must be included with all submissions.

Clinical trial registration	ClinicalTrials.gov Identifier: NCT03619512
Study protocol	Trial protocol is accessible through: ClinicalTrials.gov Identifier: NCT03619512
Data collection	Clinical data from patients were retrieved from chart review. Clinical data were collected in each center, anonymized, and securely transmitted. Data on patients were held in the main participating centers of each country (Kiel, Heidelberg, Barcelona, Nancy).
Outcomes	Outcomes were not relevant in this context, since this study is not a clinical trial, but a retrospective observational biological study.

March 2021
